# High content analysis at single cell level identifies different cellular responses dependent on nanomaterial concentrations

**DOI:** 10.1038/srep13890

**Published:** 2015-09-08

**Authors:** Bella B. Manshian, Sebastian Munck, Patrizia Agostinis, Uwe Himmelreich, Stefaan J. Soenen

**Affiliations:** 1 MoSAIC/Biomedical MRI Unit, Faculty of Medicine, KU Leuven, Herestraat 49, B3000 Leuven, Belgium; 2 Vlaams Instituut voor Biotechnologie (VIB), Center for the Biology of Disease, Leuven, Belgium; 3 Center for Human Genetics and Leuven Institute for Neuroscience and Disease (LIND), University of Leuven (KU Leuven), Leuven, Belgium; 4 Lab of Cell Death and Therapy, Faculty of Medicine, KU Leuven, Herestraat 49, B3000 Leuven, Belgium

## Abstract

A mechanistic understanding of nanomaterial (NM) interaction with biological environments is pivotal for the safe transition from basic science to applied nanomedicine. NM exposure results in varying levels of internalized NM in different neighboring cells, due to variances in cell size, cell cycle phase and NM agglomeration. Using high-content analysis, we investigated the cytotoxic effects of fluorescent quantum dots on cultured cells, where all effects were correlated with the concentration of NMs at the single cell level. Upon binning the single cell data into different categories related to NM concentration, this study demonstrates, for the first time, that quantum dots activate both cytoprotective and cytotoxic mechanisms, resulting in a zero net result on the overall cell population, yet with significant effects in cells with higher cellular NM levels. Our results suggest that future NM cytotoxicity studies should correlate NM toxicity with cellular NM numbers on the single cell level, as conflicting mechanisms in particular cell subpopulations are commonly overlooked using classical toxicological methods.

Bio-nano interactions are under continuous examination in order to further enhance the potential impact and safe use of NMs in biomedical applications^1^,^2^,^3^,^4^,^5^ to facilitate the move from pre-clinical to the clinical phase. Initially, the contribution of NM-related parameters was investigated using easily quantifiable measures such as cell viability and oxidative stress^6^,^7^. Subsequently, more mechanistic studies were being pursued, where more subtle effects such as the formation of protein coronas and the consequent effect of NMs on cellular homeostasis, the induction of lysosomal degradation pathways, such as autophagy, and the intracellular degradation of NMs were being explored^8^,^9^,^10^. Many disparate data have been generated, however, the biological impact of a certain NM-related parameter remains somewhat elusive^11^. Various explanations have been suggested for this phenomenon, including, differences between cell types^12^, incubation conditions (NM concentration, time, type of culture medium)^12^,^13^, material properties (colloidal stability, charge, size, etc.)^14^,^15^ and the type of toxicity assays performed^15^. Other factors that can contribute to this variability are the lack of adequate NM characterization and/or interference of NMs with common toxicity assays^15^,^16^. Additionally, the interactions of nanosized materials with biological components is a highly complex field, where many parameters have to be taken into account, some of which have only recently been taken into consideration. Traditionally, the induction of reactive oxygen species (ROS) and loss of cell viability are studied as main parameters for determining NM cytotoxicity^17^,^18^. Recent studies have however shown that NMs can affect cell homeostasis through a wide range of different mechanisms, for instance by induction of autophagy^9^, intracellular degradation and loss of toxic ions^19^, binding important signaling molecules (ligands/receptors) and hereby affecting both intra- and intercellular communication^20^. An important factor in bio-nano interaction studies is the formation of the protein corona around NMs. The protein corona will determine how the NM will be presented to the cells when present in physiological conditions and hereby affect the final biological outcome of cellular NM exposure^21^. Recent studies have shown that the composition of the protein corona determines where the NMs will finally end up within the cells^10^. Therefore, various methods have been set up to enable quantitative profiling of the protein coronas^22^. Much work has been put into determining the physicochemical properties of NMs and how they influence the composition of the protein corona^23^,^24^,^25^. Recently, it has also been shown that temperature plays a vital role in determining protein corona composition and cellular NM uptake^26^.

To date, NM toxicity studies are commonly performed in a manner similar to chemical toxicity studies, where for every parameter tested, a biochemical assay is used, providing a single representative value for the entire cell population. Dose-response curves are then generated by exposing cultured cells to a wide range of concentrations of NMs or chemicals. For chemicals, this has been proven to be a suitable procedure, to test their reactivity on cells, as they typically cross membranes more easily. For NMs, this procedure is more questionable as toxicity is mostly linked to the intracellular presence of NMs, apart from more rare events such as interaction with cell surface receptors or plasma membrane permeabilisation^27^. Cellular NM levels can however vary greatly and are dependent on the efficiency of endocytotic NM uptake. Various groups have therefore stressed the importance of determining cellular NM concentrations to accurately determine NM toxicity^28^,^29^,^30^, as various NM-related parameters, such as the nature of the NM coating, can influence NM toxicity as a secondary effect caused only by altered cellular NM uptake levels^28^. However, the currently used methods still link cellular effects to the average cellular NM level for the entire cell population, based, for instance, on colorimetric or inductively coupled plasma-mass spectrometry assays. Cellular NM levels have been shown to vary widely, even between closely neighboring cells^31^. Therefore, even though calculating average cellular toxicity and NM uptake levels are, to date, the most accepted methods for analyzing NM exposure yet these methods do not provide a comprehensive overview of all the processes involved in the cells of a specific population, rather they provide an average effect elicited by the NMs.

Averaging effects over a population cloaks distinct effects in multiple subpopulations. Many NM-elicited cellular responses can therefore be overlooked and only gross effects will be picked up. In order to provide a mechanistic understanding of how biological systems interact with NMs, it is therefore essential that individual cellular responses are collected. Hence, in this study, we employ a recently set up and validated methodology based on high-content imaging to evaluate a wide range of cellular responses to NM exposure^11^. We have modified the methodology to perform analysis on a single cell basis, where the consequences of exposure to fluorescent quantum dots (QDots) were correlated to the level of NM in the same cell. The data were then binned in 10 different categories, depending on the cellular QDot level, after which cellular parameters were analysed per category, to represent a substantial subcategory of the entire cellular population. At the same time, analysis was also performed without taking cellular NM levels into account, where the data provided by the two analysis methods can be immediately compared to one another.

## Materials and Methods

### Cell culture

Mouse embryonic fibroblasts (MEFs) and transgenic MEFs (MEF Atg5 KO and MEF Bax/Bak DKO) were grown in high glucose containing Dulbecco’s modified Eagle’s medium (DMEM), supplemented with 10% fetal calf serum, 1 mM sodium pyruvate, 2 mM L-Glutamine and 1% penicillin/streptomycin (Gibco, Invitrogen, Belgium). The MEF cells were passaged upon reaching 80% confluence and reseeded at a ratio of 1:5.

Mouse mesenchymal stem cells were maintained in high glucose containing Dulbecco’s modified Eagle’s medium (DMEM), supplemented with 10% fetal calf serum, 10% horse serum, 1 mM sodium pyruvate and 2 mM L-Glutamine (Gibco, Incitrogen, Belgium). Cells were passaged when reaching nearly 80% confluence and reseeded at a density of 100,000 cells/flask in 75 cm^2^ tissue culture flasks (Nunc, Belgium).

### Cell nanoparticle interaction studies

For high-content imaging studies, all cell types were seeded at 7500 cells/well in a 24 well plate (Nunc, Belgium). Cells were allowed to attach overnight in a humidified atmosphere at 37 °C and 5% CO_2_, after which the cells were incubated with the carboxylated QDots for 24 h in full growth medium. For cellular exposure studies, cells were incubated with the QDots at 2.5, 5, 7.5, 10, 12.5, 15 or 20 nM. For intracellular QDot distribution studies, MSCs were incubated with the QDots at 10 nM, while MEF cells were incubated with 12.5 nM QDots. For mechanistic studies, MEF, MEF Atg 5 KO or MEF Bax/Bak DKO cells were used and incubated with the QDots for 24 h at 12.5 nM. MSCs were either exposed to the QDots directly (10 nM) or were co-incubated with autophagy inhibitor 3-methyladenine (3-MA; 1 mM; Sigma-Aldrich, Belgium) or pan-caspase inhibitor Z-VAD-fmk (100 μM, Bachem AG, Switzerland). Each experiment was performed in three independent repeats and data were analyzed using full data sets of the different repeats. The specific experimental procedures for the high-content imaging experiments are described in the Supplementary Information.

### Statistical analysis

All data are expressed as the mean + standard error to the mean (SEM). For all experiments, any statistical significance between a single condition and untreated control cells were analyzed using the *t*-test statistical method.

## Results

### Average effects of quantum dots on cultured cells

**Figure 1:**
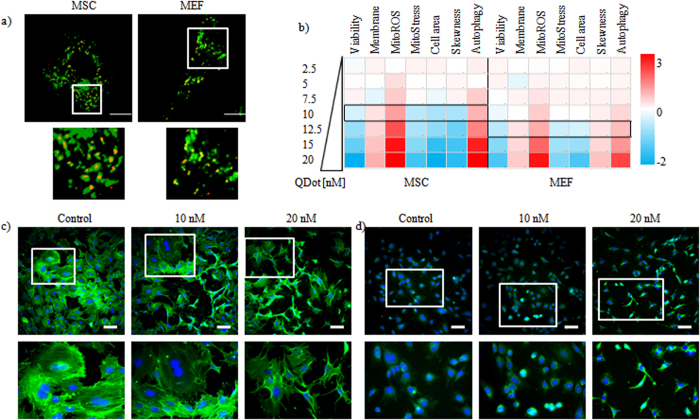
Effects of exposure to varying concentrations of QDots in MSCs and MEFs. (**a**) representative confocal micrographs of MSC (left) and MEF (right) cells that were transduced with CellLight Lysosomes-GFP (green) and subsequently exposed to 10 nM QDots (red) for 24 h. yellow/orange dots represent colocalized QDots and lyosomes. Scale bars = 25 μm. The area in the white rectangle is shown as a magnified view below the image. (**b**) Heat maps of high-content imaging-based data for MSCs and MEFs exposed to varying concentrations of QDots and analyzed for; cell viability, cell membrane damage, mitochondrial ROS, size of the mitochondrial network, area of the cell, skewness of the cell, and level of autophagy. Data are shown as relative values after z-normalization compared to untreated control cells (=1) where the fold-change is indicated by the respective color-code. Data have been acquired for minimum 5000 cells/condition which were gathered from three independent experiments. (**c,d**) Representative InCell high-content images of control MSCs or MSCs exposed to the QDots at 10 or 20 nM for 24 h, after which the cells were stained for (**c**) β-actin (green) or (**d**) mitochondrial ROS (green). Cells were counterstained with Hoechst nuclear stain (blue). Scale bars = 100 μm, the area in the white rectangle is depicted in a magnified view below the original image.

The QDots used are commercially available CdSe/ZnS core/shell nanoparticles (Invitrogen, Belgium) with maximal emission at 655 nm, which were coated using a carboxylated amphiphilic polymer, generating negatively charged NPs (Supp Section S1). When exposing mouse mesenchymal stem cells (MSCs) and mouse embryonic fibroblasts (MEFs) to these QDots, a clear endosomal localization can be observed, as the QDots are efficiently endocytosed and do not merely adhere to the plasma membrane (Fig. 1a, Supp Fig. S2), which is in line with other reports on carboxylated QDots^28^. Next, MSCs and MEFs were exposed to a series of QDot concentrations, and the effect on various parameters was evaluated (Fig. 1b–d). The data reveal significant concentration-dependent effects of the QDots on cell viability (Supp Fig. S3), cell membrane damage (Supp Fig. S4), autophagy induction (Supp Fig. S5), cell morphology (Supp Fig. S6), cell skewness (Supp Fig. S7), size of the mitochondrial network (Supp Fig. S8) and mitochondrial ROS (Supp Fig. S9), at concentrations of 15 nM or more. These values are in line with previous studies using carboxylated QDots^28^, suggesting that QDots appear to affect cell homeostasis through various mechanisms.

### Effects of quantum dots on cultured cells at the single cell level

**Figure 2:**
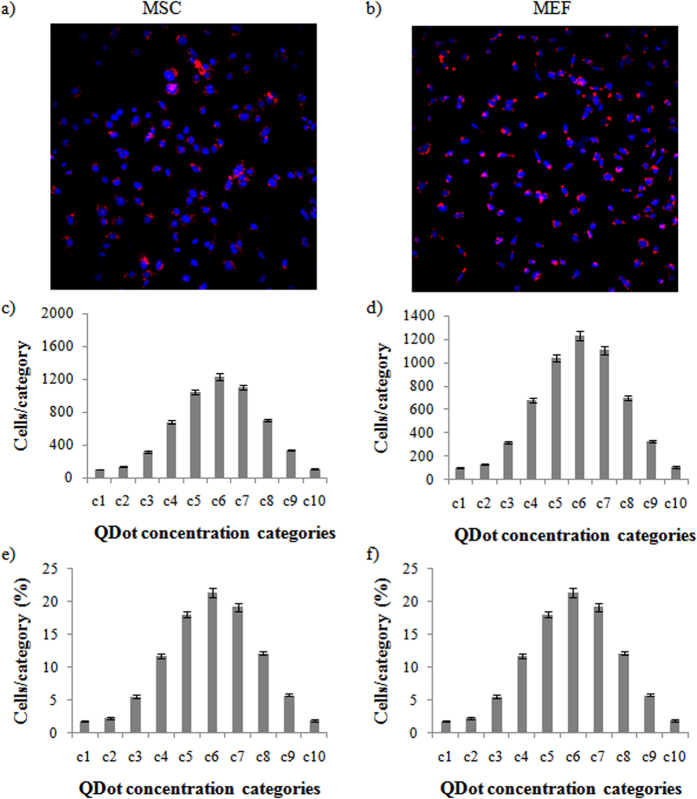
Cellular distribution of cell-associated fluorescent quantum dots. (**a,b**) Representative images of (**a**) MSCs exposed to 10 nM QDots for 24 h or (**b**) MEFs exposed to 12.5 nMQDots for 24 h. (**c,d**) Histograms presenting the number of (**c**) MSC or (**d**) MEF cells per category when the population is divided into 10 categories spanning the entire range of cellular QDot levels. (**e,f**) Histograms presenting the percentage of (**e**) MSC or (**f**) MEF cells per category for the total population analyzed.

When considering average results over the entire visualized population, our data reveal that the effects the QDots induce in MSCs at 10 nM are similar to those observed in MEFs at 12.5 nM. At these concentrations, no significant effects could be observed, apart from the induction of oxidative stress, which does not appear to induce any cell death at these levels due to natural cellular defense mechanisms^32^. Exposure of both cell types to the QDots (MSC at 10 nM; MEF at 12.5 nM) however resulted in a broad distribution in cellular QDot levels (Fig. 2), as is typical for *in vitro* cultured cells exposed to NMs^31^. NM levels between the two cell types were highly similar which explains the high similarity in their toxicity profiles. In order to evaluate the impact of cellular NM distribution on its resulting toxicity, the fluorescent properties of the QDots were exploited to determine relative cellular NM levels, and consequently every cellular parameter was linked to a subpopulation of cells with a certain NM level. NM distribution in cells followed a near-Gaussian profile and cells were subdivided into 10 different categories, category 1 being the lowest NM level and category 10 the highest (Fig. 2c–f). The data reveal that the majority of cells are in the medium categories (c4–c7; 68%). The lower categories (c1–c3) represent merely 12% of the entire population while the higher categories (c8–c10) represent 20% of the population. Interestingly, for MSCs exposed to 10 nM QDots and MEFs exposed to 12.5 nM QDots, the distribution of cellular QDot levels is quite similar, suggesting that the high similarity in cellular effects under these conditions (Fig. 1) is due to the similar levels of intracellular QDots. In general, MEF cells appear to be slightly more resistant to QDot-elicited cytotoxic effects as a secondary mechanism caused by the intrinsic lower internalization efficiency of these cells.

**Figure 3:**
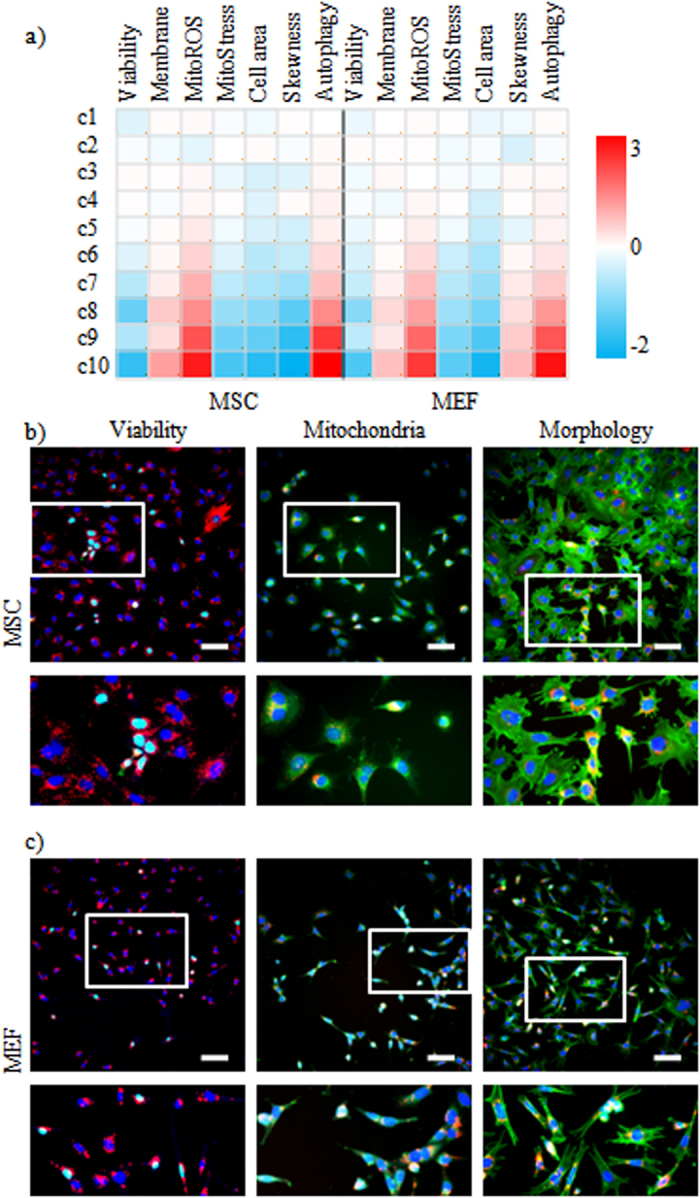
Overview of cellular effects in view of cellular QDot concentration at “non-toxic” conditions. (**a**) Heat maps of high-content imaging-based data for MSCs and MEFs exposed to QDots at 10 and 12.5 nM concentrations, respectively, at which slight but non-significant effects had been observed for nearly all the tested parameters based on the total cell population. Cells were analyzed for; cell viability, cell membrane damage, mitochondrial ROS, size of the mitochondrial network, area of the cell, skewness of the cell, and level of autophagy. For every cell, the relative level of QDots was also calculated based on the cellular QDot intensity and area, after which cells were divided into 10 categories based on their cellular QDot levels, ranging from c1 (lowest) to c10 (highest). Observed cellular effects were grouped based on the different categories of intracellular QDot concentrations. The data are shown as relative values after z-normalization compared to untreated control cells (=1) where the fold-change is indicated by the respective color-code. Data were acquired for minimum 5000 cells/condition and were gathered from three independent experiments. (**b,c**) Representative InCell high-content images of (**b**) MSCs and (**c**) MEFs exposed to the QDots at 10 and 12.5 nM, respectively. Red: QDots, Blue: Hoechst nuclear stain, Green: dead cells (left), mitochondrial ROS (middle), β-actin (right). Scale bars = 100 μm, the area in the white rectangle is depicted in a magnified view below the original image.

Following this categorization, the results showed a clear impact of cellular NM levels on both the nature and degree of the cellular response (Fig. 3). For cells containing low to medium QDot levels, no significant effects were observed for any of the assessed parameters. However, at higher intracellular NM levels (categories 8–10), significant effects were observed, which, however, did not all correlate with intracellular NM levels. Mitochondrial stress and ROS (Supp Figs S09, S10), cell morphology and skewness (Supp Figs S11, S12) and autophagy (Supp Fig. S13) showed higher significance with an increase in intracellular QDot levels, whereas the highest levels of cell death and associated membrane damage were seen in categories 8 and 10, but less pronounced in category 9 (Supp Figs S14, S15).

### Mechanistic understanding of quantum dot-induced cytotoxic effects at single cell level

**Figure 4:**
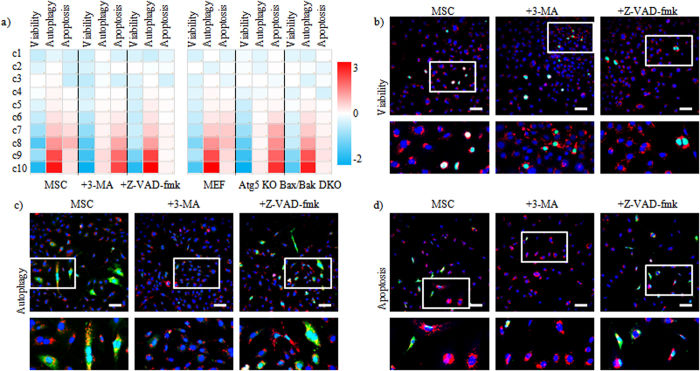
Overview of conflicting mechanisms involved in general cellular response. (**a**) Heat maps of high-content imaging-based data for MSCs, MSCs treated with 3-MA or Z-VAD-fmk or MEFs, MEF Atg5 KO or MEF Bax/Bak DKO exposed to QDots at 10 nM (MSC cells) and 12.5 nM (MEF cells), respectively. Cells were analyzed for; cell viability, autophagy and apoptosis. For every cell, the relative level of QDots was also calculated based on the cellular QDot intensity and area, after which cells were divided into 10 categories based on their cellular QDot levels, ranging from c1(lowest) to c10 (highest). After analysis, the cellular effects were grouped based on the different categories for cellular QDot concentrations. The data are shown as relative values after z-normalization compared to untreated control cells (=1) where the fold-change is indicated by the respective color-code. Data were acquired for minimum 5000 cells/condition and were gathered from three independent experiments. (**b**–**d**) Representative InCell high-content images of MSCs, MSCs treated with 3-MA or MSC Z-VAD-fmk exposed to QDots (10 nM) for 24 h, after which the cells were stained for (**b**) cell death, (**c**) autophagy (LC3) and (**d**) apoptosis (active caspase-3). Red: QDots, Blue: Hoechst nuclear stain, Green: (**b**) dead cells, (**c**) LC3 (**d**) active caspase-3. Scale bars = 100 μm, the area in the white rectangle is depicted in a magnified view below the original image.

NM-mediated autophagy has been described to be a general cellular response to exposure to a wide range of NMs, including QDots^9^. The physiological impact of NM-induced autophagy remains however somewhat unclear, as some studies have indicated clear cytoprotective effects by impeding cellular apoptosis signaling, while other studies showed direct autophagy-mediated cell death^33^,^34^. The induction of autophagy was therefore selected as the potential key mechanism to explain the lower correlation of intracellular NM levels and cell death. We tested two transgenic MEF cell lines, displaying either compromised autophagy, because of the deletion of the essential autophagy gene Atg5 (*i.e.* MEF-Atg5 KO) or mitochondrial apoptosis due to the double deficiency in pro-apoptotoc Bax and Bak (*i.e.* MEF-Bax/Bak DKO), a defect known to blunt apoptosis to a variety of stress signals^35^,^36^. Along with these genetic approaches, we chemically inhibited autophagy (3-methyladenine (3-MA)) and apoptosis (Z-VAD-fmk) in MSCs. Figure 4 reveals clear involvement of apoptosis and autophagy in QDot-mediated cellular response. Inhibition of apoptosis did not affect the levels of cellular autophagy (Supp Fig. S16), whereas inhibition of autophagy resulted in a clear increase in cellular apoptosis levels at higher intracellular QDot concentrations (Supp Fig. S17). Interestingly, inhibition of either mechanism could not prevent cell death, yet in apoptosis-deficient cells, cell death only occurred at the highest intracellular QDot concentration, whereas in autophagy-deficient cells, cell death induction followed a clear concentration-dependent profile (Supp Fig. S18).

## Discussion

In the present study, we have demonstrated a clear impact of intracellular NM concentration and NM distribution throughout the entire cell population on the cellular response to NM exposure. Upon using biochemical assays that commonly generate mean values for an entire cell population, more subtle effects that are only present in a certain subpopulation of the cells are normally overlooked, although they can have serious consequences and limitations for the final use of the cells. Biochemical assays are often used as they are generally easy to perform, amenable to high-throughput and quantitative. Imaging-based methods provide visual information on the impact of NMs, and are therefore generally qualitative. Some parameters, such as cell morphology can however only be properly studied using visual analysis. To overcome these limitations, several research groups have turned to the use of high content imaging^37^. One of the main advantages of high content imaging is the automated generation of a high number of images that can then be analyzed and turned into quantitative information over thousands of cells per condition^38^. The use of high content imaging has gained high interest from many fields, including cell biology and drug discovery as a powerful quantitative method that enables the generation of multidimensional (multiple parameters) and hierarchical data (*i.e*. in this study: all parameters linked to cellular QDot levels)^38^. Additional advantages of high content imaging are the ability to discriminate between real and off-target effects, non-specific fluorescence (*i.e*. interference of NMs), or the ability to study additional parameters (*i.e.* NM distribution).

The results obtained in this study are in line with previous reports on the toxicity of CdSe/ZnS QDots, coated with amphiphilic polymers, where clear effects on cell viability could be observed at concentrations ranging from about 2–20 nM^28^,^39^,^40^. This wide range of safe upper concentrations is mainly linked with the generation of disparate data due to differences in cell types, QDot surface chemistry and analysis method used. For cadmium-containing QDots, their toxicity has been mainly associated with the presence of free Cd^2+^ ions, that are known toxic agents. The presence of Cd^2+^ ions can result from remnants of cadmium after the synthesis and purification, where in a hydrophilic oxidative environment, some Cd^2+^ will also leach from the QDot surface, as a result of the inequilibrium of Cd^2+^ ions in solution and within the QDots. In a cellular environment, the QDots will be exposed to the degradative acidic microenvironment of the endosomes in which they will locate, which will further increase the loss of Cd^2+^ ions due to acid etching of the surface^19^,^28^,^41^. The present work shows that the second mechanism is more predominant in affecting cellular viability, as toxicity is clearly linked to intracellular QDot levels and thus Cd^2+^ ions being released intraendosomally. If toxicity was caused by free Cd^2+^ ions in solution, then any toxic effects would be more homogeneously spread and not linked so closely to the intracellular QDot levels. This of course also depends on the quality and purity of the QDot sample, where “aged” QDots or QDots that have been poorly purified and contain high Cd^2+^ levels in the stock suspension, may be more prone to toxicity induced by the higher levels of free Cd^2+^ ions^42^.

A clear difference between the two cell types in terms of their ability to deal with NM exposure has been observed. The MSCs and MEFs show similar effects when exposed to 10 and 12.5 nM QDots, respectively. This has then be shown to result in highly similar intracellular levels of the QDots, thus showing that the intracellular level is more meaningful in terms of comparing different cell types than the concentration of NMs used for initial cell exposure. To date, nearly all studies however express NM toxicity in terms of exposure concentrations, which, as shown here, is less meaningful. The difference in cellular NM concentrations between different cell types can be due to a lower intrinsic endocytic capacity of one cell type compared to the other, or a smaller surface area and hence less potential interaction area for NMs with the cell surface. Other studies have made similar observations, where the concept of “cell vision” has been introduced as a key factor in nano-bio interactions. In short, “cell vision” signifies the intrinsic capacity of a particular cell type to deal with NMs, which is determined by the cell-specific uptake and defense mechanisms^43^,^44^.

For mechanistic studies, the overall effect of the NMs will dominate, which requires high NM concentrations. By linking cellular responses to the intracellular NM concentration on the single cell level, we have shown that at apparent sub-cytotoxic NM incubation concentrations, various conflicting mechanisms are evoked, such as apoptosis and autophagy. Taken together, these data suggest that apoptosis occurs at medium to high intracellular QDot concentrations, but is then impeded by autophagy at higher concentrations, resulting in a partial recovery of cell viability. At the highest intracellular QDot concentrations, autophagy levels were too high, resulting in autophagy-mediated cell death. One possibility is that the level of Cd^2+^ ions present in the QDot incubation media, are responsible for the induction of apoptosis as the release of Cd^2+^ has been shown to be a major cause of QDot toxicity^41^. Particularly for cell-internalized QDots, degradation has been observed to occur at a higher rate, resulting in the generation of free Cd^2+^ ions. Cd^2+^ ions are known to be highly toxic and a sustained cellular exposure to high levels of these ions (partially derived from the initial incubation mixture and partially from intracellular QDot dissolution) will result in cell death, through the process of apoptosis. The precise mechanism underlying the induction of autophagy remains somewhat unclear, but oxidative stress-induced mitochondrial damage, which is known to result in a particular form of autophagy, called mitophagy^45^, could be involved. Autophagy in itself is known to be a cytoprotective mechanism^46^ and induction of autophagy is known to inhibit apoptosis signaling^47^. Many different types of NMs, including various types of Cd^2+^− containing ones, have been shown to induce autophagy, which either results in cytotoxicity or cytoprotection^33^,^34^,^48^. Our findings reveal a clear induction of apoptosis for higher levels of cellular NMs, which is however inhibited by the concurrent induction of autophagy. Together, this results in variable levels of cell death, where at low levels of autophagy, apoptosis is more pronounced and cell viability decreases. When autophagy levels increase, apoptosis is more inhibited, resulting in a recovery of cell viability. At high autophagy levels, cell viability again decreases, although apoptosis is still strongly inhibited. These data suggest that at the highest levels of autophagy, cell death occurs through direct autophagy-mediated signaling^49^.

These findings can explain the high level of disparate data that have been generated on NM toxicity, where small differences in cell types, exposure conditions or NM properties can result in changes in cellular NM levels, and hereby affect the balance between the different cellular responses. We have, for the first time, demonstrated a robust method to evaluate NM toxicity on high cell numbers with single cell resolution, allowing to delineate cellular responses in specific cellular subpopulations. The data here demonstrate the clear need for correlating cellular responses with intracellular NM concentration to enhance our understanding of NM toxicity and the precise mechanisms by which NMs can interact with biological components. Additionally, the findings reveal that data obtained from average population analyses overestimate the safe concentrations of NMs, due to the added cellular variability. Although single cell analysis was achieved, binning the data into the different categories provided a nicer and more achievable overview of substantial parts of the entire cell population, as revealed by the data in this study where significant effects are observed in nearly 20% of the entire population, but this goes unnoticed in the entire cell population. This knowledge is imperative for the future progress and clinical translation of nanotechnology and the methodology demonstrated here can serve as a template for future nanotoxicological analyses.

## Additional Information

**How to cite this article**: Manshian, B. B. *et al.* High content analysis at single cell level identifies different cellular responses dependent on nanomaterial concentrations. *Sci. Rep.* **5**, 13890; doi: 10.1038/srep13890 (2015).

## Supplementary Material

Supplementary Information

## References

[b1] Hansen S. F., Maynard A., Baun A. & Tickner J. A. Late lessons from early warnings for nanotechnology. Nature Nanotech. 3, 444–447 (2008). 10.1038/nnano.2008.198 18685623

[b2] Cho E. C., Zhang Q. & Xia Y. N. The effect of sedimentation and diffusion on cellular uptake of gold nanoparticles. Nature Nanotech. 6, 385–391 (2011). 10.1038/nnano.2011.58 PMC3227810 21516092

[b3] Kim J. A., Aberg C., Salvati A. & Dawson K. A. Role of cell cycle on the cellular uptake and dilution of nanoparticles in a cell population. Nature Nanotech. 7, 62–68 (2012). 10.1038/nnano.2011.191 22056728

[b4] Alkilany A. M. & Murphy C. J. Toxicity and cellular uptake of gold nanoparticles: what we have learned so far? J Nanopart. Res. 12, 2313–2333 (2010). 21170131 10.1007/s11051-010-9911-8 PMC2988217

[b5] Medina C., Santos-Martinez M. J., Radomski A., Corrigan O. I. & Radomski M. W. Nanoparticles: pharmacological and toxicological significance. Brit. J. Pharmacol. 150, 552–558 (2007). 17245366 10.1038/sj.bjp.0707130 PMC2189773

[b6] Braydich-Stolle L., Hussain S., Schlager J. J. & Hofmann M. C. *In vitro* cytotoxicity of nanoparticles in mammalian germline stem cells. Toxicol. Sci. 88, 412–419 (2005). 16014736 10.1093/toxsci/kfi256 PMC2911231

[b7] Lai J. C. *et al.* Exposure to titanium dioxide and other metallic oxide nanoparticles induces cytotoxicity on human neural cells and fibroblasts. Int. J.Nanomed. 3, 533–545 (2008). 10.2147/ijn.s3234 PMC2636591 19337421

[b8] Lynch I., Salvati A. & Dawson K. A. Protein-nanoparticle interactions what does the cell see? Nature Nanotech. 4, 546–547 (2009). 10.1038/nnano.2009.248 19734922

[b9] Peynshaert K. *et al.* Exploiting intrinsic nanoparticle toxicity: the pros and cons of nanoparticle-induced autophagy in biomedical research. Chem. Rev. 114, 7581–7609 (2014). 24927160 10.1021/cr400372p

[b10] Walkey C. D. *et al.* Protein corona fingerprinting predicts the cellular interaction of gold and silver nanoparticles. ACS Nano 8, 2439–2455 (2014). 24517450 10.1021/nn406018q

[b11] Manshian B. B. *et al.* High-content imaging and gene expression analysis to study cell-nanomaterial interactions: The effect of surface hydrophobicity. Biomaterials 35, 9941–9950 (2014). 25218858 10.1016/j.biomaterials.2014.08.031

[b12] Kim I. Y., Joachim E., Choi H. & Kim K. Toxicity of silica nanoparticles depends on size, dose, and cell type. Nanomedicine 11, 1407–1416 (2015). 25819884 10.1016/j.nano.2015.03.004

[b13] Maiorano G. *et al.* Effects of cell culture media on the dynamic formation of protein-nanoparticle complexes and influence on the cellular response. ACS Nano 4, 7481–7491 (2010). 21082814 10.1021/nn101557e

[b14] Yoon D. *et al.* Agglomeration, sedimentation, and cellular toxicity of alumina nanoparticles in cell culture medium. *J. Nanopart. Res*. 13, 2543–2551 (2011).

[b15] Rivera Gil P., Oberdorster G., Elder A., Puntes V. & Parak W. J. Correlating physico-chemical with toxicological properties of nanoparticles: the present and the future. ACS Nano 4, 5527–5531 (2010). 20973573 10.1021/nn1025687

[b16] Ong K. J. *et al.* Widespread nanoparticle-assay interference: implications for nanotoxicity testing. Plos One 9, (2014). 10.1371/journal.pone.0090650 PMC3949728 24618833

[b17] Lin W., Huang Y. W., Zhou X. D. & Ma Y. *In vitro* toxicity of silica nanoparticles in human lung cancer cells. Toxicol. Appl. Pharmacol. 15, 252–259 (2006). 17112558 10.1016/j.taap.2006.10.004

[b18] Liu S., Xu L., Zhang T., Ren G. & Yang. Z. Oxidative stress and apoptosis induced by nanosized titanium dioxide in PC12 cells. Toxicology 267, 172–177 (2010). 19922763 10.1016/j.tox.2009.11.012

[b19] Soenen S. J., Rejman J., Parak W. J. & Manshian B. (Intra) cellular stability of inorganic nanoparticles: effects on cytotoxicity, particle functionality, and biomedical applications. Chem. Rev. 115, 2109–2135 (2015). 25757742 10.1021/cr400714j

[b20] Comfort K. K., Maurer E. I., Braydich-Stolle L. K. & Hussain S. M. Interference of silver, gold, and iron oxide nanoparticles on epidermal growth factor signal transduction in epithelial cells. ACS Nano 5, 10000–10008 (2011). 22070748 10.1021/nn203785a

[b21] Nazarenus M. *et al.* *In vitro* interaction of colloidal nanoparticles with mammalian cells: What have we learned thus far? Beilstein J. Nanotechnol. 5, 1477–1490 (2014). 25247131 10.3762/bjnano.5.161 PMC4168913

[b22] Docter D. *et al.* Quantitative profiling of the protein coronas that form around nanoparticles. Nature Protoc. 9, 2030–2044 (2014). 25079427 10.1038/nprot.2014.139

[b23] Lundqvist M. *et al.* The evolution of the protein corona around nanoparticles: a test study. ACS Nano 5, 7503–7509 (2011). 21861491 10.1021/nn202458g

[b24] Rocker C., Potzl M., Zhang F., Parak W. J. & Nienhaus G. U. A quantitative fluorescence study of protein monolayer formation on colloidal nanoparticles. Nat. Nanotechnol. 4, 577–580 (2009). 19734930 10.1038/nnano.2009.195

[b25] Mahmoudi M. *et al.* Irreversible changes in protein conformation due to interaction with superparamagnetic iron oxide nanoparticles. Nanoscale 3, 1127–1138 (2011). 21210042 10.1039/c0nr00733a

[b26] Mahmoudi M. *et al.* Temperature: The “ignored” factor at the nanobio interface. ACS Nano 7, 6555–6562 (2013). 23808533 10.1021/nn305337c

[b27] Turabekova M. *et al.* Immunotoxicity of nanoparticles: a computational study suggests that CNTs and C60 fullerenes might be recognized as pathogens by Toll-like receptors. Nanoscale 6, 3488–3495 (2014). 24548972 10.1039/c3nr05772k

[b28] Soenen S. J., Demeester J., De Smedt S. C. & Braeckmans K. The cytotoxic effects of polymer-coated quantum dots and restrictions for live cell applications. Biomaterials 33, 4882–4888 (2012). 22494884 10.1016/j.biomaterials.2012.03.042

[b29] Chang E., Thekkek N., Yu W. W., Colvin V. L. & Drezek R. Evaluation of quantum dot cytotoxicity based on intracellular uptake. Small 2, 1412–1417 (2006). 17192996 10.1002/smll.200600218

[b30] Ketkar-Atre *et al.* Variability in contrast agent uptake by different but similar stem cell types. *Int. J. Nanomed*. 8, 4577–4591 (2013). 10.2147/IJN.S51588 PMC3876490 24399873

[b31] Summers H. D. *et al.* Statistical analysis of nanoparticle dosing in a dynamic cellular system. Nature Nanotech. 6, 170–174 (2011). 10.1038/nnano.2010.277 21258333

[b32] Nel A., Xia T., Madler L. & Li N. Toxic potential of materials at the nanolevel. Science 311, 622–627 (2006). 16456071 10.1126/science.1114397

[b33] Andon F. T. & Fadeel B. Programmed cell death: molecular mechanisms and implications for safety assessment of nanomaterials. Acc. Chem. Res. 46, 733–742 (2013). 22720979 10.1021/ar300020b

[b34] Luo Y. H. *et al.* Cadmium-based quantum dot induced autophagy formation for cell survival via oxidative stress. Chem. Res. Toxicol. 26, 662–673 (2013). 23617821 10.1021/tx300455k

[b35] Lin C. W., Jan M. S. & Kuo J. H. Autophagy-related gene expression analysis of wild-type and atg5 gene knockout mouse embryonic fibroblast cells treated with polyethylenimine. Mol. Pharm. 11, 3002–3008 (2014). 25075440 10.1021/mp500111u

[b36] Liu J. *et al.* A diterpenoid derivate compound targets selenocysteine of thioredoxin reductases and induces Bax/Bak-independent apoptosis. Free Radic. Biol. Med. 63, 485–494 (2013). 23732520 10.1016/j.freeradbiomed.2013.05.038

[b37] Brayden D. J., Cryan S. A., Dawson K. A., O’Brien P. J. & Simpson J. C. High-content analysis for drug delivery and nanoparticle applications. Drug Discovery Today (2015), 10.1016/j.drudis.2015.04.001. 25908578

[b38] Singh S., Carpenter A. E. & Genovesio A. Increasing the content of high-content screening: An overview. *J. Biomol. Screen*. 19, 640–650 (2014). 24710339 10.1177/1087057114528537 PMC4230961

[b39] Kirchner C. *et al.* Cytotoxicity of colloidal CdSe and CdSe/ZnS nanoparticles. Nano Lett. 5, 331–338 (2005). 15794621 10.1021/nl047996m

[b40] Soenen S. J. *et al.* The effect of nanoparticle degradation on amphiphilic polymer-coated quantum dot toxicity: the importance of particle functionality assessment in toxicology. *Acta Biomater*. 10, 732–741 (2014). 24121195 10.1016/j.actbio.2013.09.041

[b41] Derfus A. M., Chan W. C. W. & Bhatia S. N. Probing the cytotoxicity of semiconductor quantum dots. Nano Lett. 4, 11–18 (2004). 10.1021/nl0347334 PMC5588688 28890669

[b42] Gagné F., Maysinger D., André C. & Blaise C. Cytotoxicity of aged cadmium-telluride quantum dots to rainbow trout hepatocytes. Nanotox. 2, 113–120 (2008).

[b43] Mahmoudi M. *et al.* Cell “vision”: complementary factor of protein corona in nanotoxicology. Nanoscale 4, 5461–5468 (2012). 22842341 10.1039/c2nr31185b

[b44] Xue J. *et al.* An assessment of the impact of SiO2 nanoparticles of different sizes on the rest/wake behavior and the developmental profile of zebrafish larvae. Small 9, 3161–3168 (2013). 23468419 10.1002/smll.201300430

[b45] Mishra P. & Chan D. C. Mitochondrial dynamics and inheritance during cell division, development and disease. Nat. Rev. Mol. Cell. Bio. 15, 634–646 (2014). 25237825 10.1038/nrm3877 PMC4250044

[b46] Yang Z., Goronzy J. J. & Weyand C. M. Autophagy in autoimmune disease. J. Mol. Med. (Berl.) 93, 707–717 (2015). 26054920 10.1007/s00109-015-1297-8 PMC4486076

[b47] Liu D., Gao M., Yang Y., Qi Y. U., Wu K. & Zhao S. Inhibition of autophagy promotes cell apoptosis induced by the proteasome inhibitor MG-132 in human esophageal squamous cell carcinoma EC9706 cells. Oncol. Lett. 9, 2278–2282 (2015). 26137056 10.3892/ol.2015.3047 PMC4467331

[b48] Wang Q. W. *et al.* Cadmium-induced autophagy promotes survival of rat cerebral cortical neurons by activating class III phosphoinositide 3-kinase/beclin-1/B-cell lymphoma 2 signaling pathways. Mol. Med. Rep. 12, 2912–2918 (2015). 25955216 10.3892/mmr.2015.3755

[b49] Pi H. *et al.* SIRT3-SOD2-mROS-dependent autophagy in cadmium-induced hepatotoxicity and salvage by melatonin. Autophagy 11, 1037–1051 (2015). 26120888 10.1080/15548627.2015.1052208 PMC4590599

