# Supplementary material for: High content analysis at single cell level identifies different cellular responses dependent on nanomaterial concentrations
Source: Sci Rep. 2015 Sep 8;5:13890. doi: 10.1038/srep13890 (PMC4561960; doi:10.1038/srep13890)
Supplement: Supplementary Information [file srep13890-s1.pdf]

High content analysis at single cell level identifies different cellular responses dependent on nanomaterial concentrations.

Bella B. Manshian[1], Sebastian Munck[2,3], Patrizia Agostinis[4], Uwe Himmelreich[1], Stefaan J. Soenen[1,*]

[1]MoSAIC/Biomedical MRI Unit, Faculty of Medicine, KU Leuven, Herestraat 49, B3000 Leuven, Belgium

[2]Vlaams Instituut voor Biotechnologie (VIB), Center for the Biology of Disease, Leuven, Belgium

[3]Center for Human Genetics and Leuven Institute for Neuroscience and Disease (LIND), University of Leuven (KU Leuven), Leuven, Belgium

[4]Lab of Cell Death and Therapy, Faculty of Medicine, KU Leuven, Herestraat 49, B3000 Leuven, Belgium

* Corresponding author: Email: Stefaan.Soenen@med.kuleuven.be

Tel: +32 16 330034

Supplementary information

Supplementary section 1: Nanoparticle characteristics.

In the present study, we selected commercially available quantum dots (QDots), from a well-established supplier (QD655 ITK carboxyl; Invitrogen – Quantum Dot Corporation). These QDots have been frequently used in other studies and have been shown to display well-defined and reproducible characteristics. The QDots are CdSe/ZnS core-shell nanoparticles with an ellipsoidal shape, with a minor axis of 6 nm and a major axis of 12 nm (Quantum Dot Corporation, 2005). The QDots are obtained as a sterile 8 µM suspension in 50 mM borate buffer, pH 9.0. In this buffer, the hydrodynamic diameter of the particles is 18 nm (Quantum Dot Corporation).

In our own hands, the hydrodynamic diameter and ζ-potential of the QDots suspended in phosphate buffered saline (PBS, 10 mM, pH 7.0) were determined to be 23.4 $\pm$ 3.8 nm (polydispersity index: 0.201) and the ζ-potential was - 24.2 $\pm$ 4.9 mV. These measurements were performed using a Nanosizer instrument (Malvern, Worcestershire, UK), where 10 cycles per run were performed in triplicate. For the hydrodynamic diameter of the particles, the intensity scaling was used.

Supplementary section 2: Cellular quantum dot internalization.

The cellular internalization of the QDots by MSCs and MEFs were examined using confocal microscopy, where z-stacks of cells exposed to the QDots (10 nM for MSCs and 12.5 nM for MEFs) were acquired after which the cells were stained for α-tubulin as a cytoplasmic marker. It was observed that the QDots were efficiently internalized by the cells as no particles or agglomerates could be seen on top of the cell surface, but all quantum dot-associated signals originated from the middle slices.

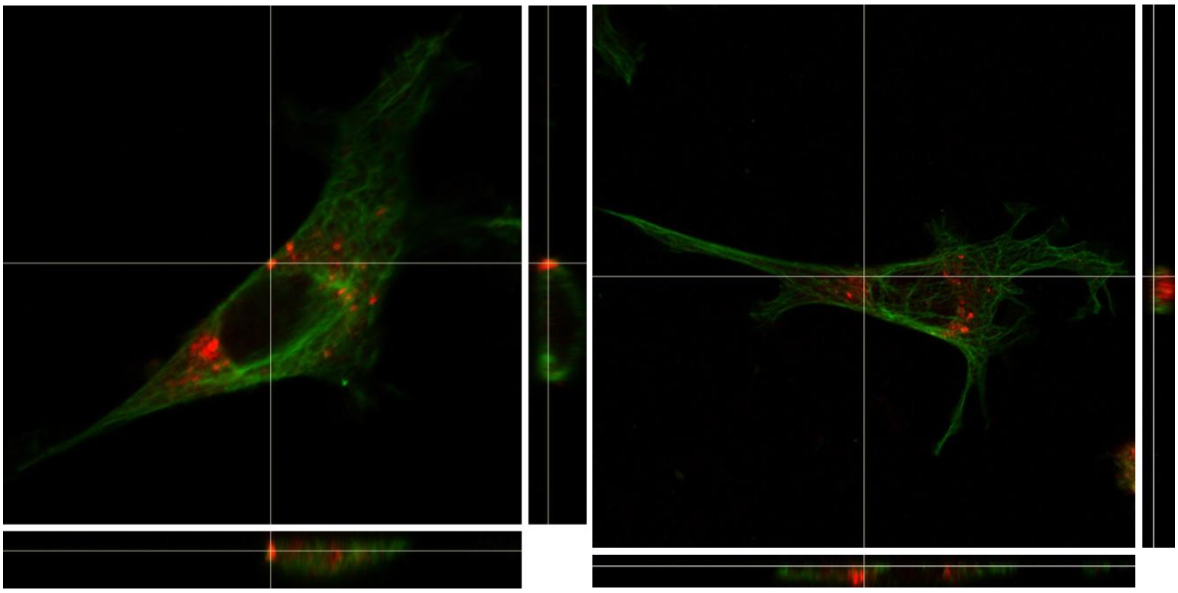

MSC MEF

Figure S1. Confocal micrographs of MSC (left) and MEF (right) cells exposed to the QDots for 24 h at 10 or 12.5 nM, respectively, followed by fixation of the cells and immunostaining for α-tubulin (green). The z-stacked images are shown in orthogonal view, to enable a clear visualization of quantum dot distribution throughout the cell.

Supplementary section 3: Cellular effects induced by varying quantum dot concentrations.

To evaluate the effects of the QDots on cellular homeostasis, various parameters were analyzed for both the MSC or MEF cells exposed to 2.5, 5, 7.5, 10, 12.5, 15 or 20 nM QDots for 24 h. All experiments were repeated three times and the data were collected for a minimum of 5000 cells/condition. Here, histograms are shown of the high content imaging-derived data where values are presented as relative values respective to untreated control cells (= 1) as mean ± SEM (n = 3). The histograms are shown per cellular parameter. Shown below are the data for cell viability.

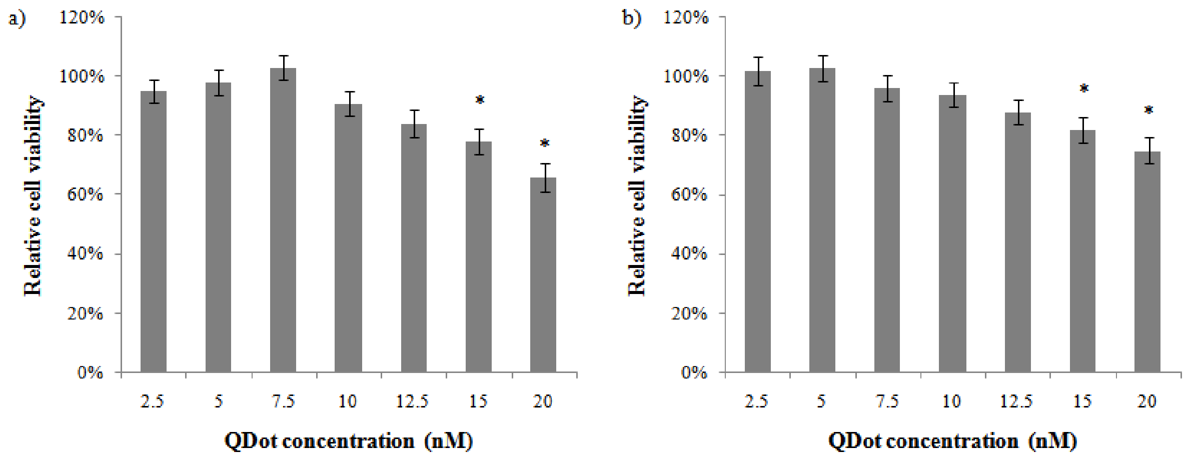

Figure S2. Histograms representing the relative cell viability of (a) MSC and (b) MEF cells exposed to the QDots at 2.5, 5, 7.5, 10, 12.5, 15 or 20 nM for 24 h. Data are shown as relative values normalized to untreated control cells (= 100%) as mean ± SEM (n = 3). The degree of statistical significance is indicated where appropriate (*: $p \leq 0.05$).

Shown below are the data for membrane damage.

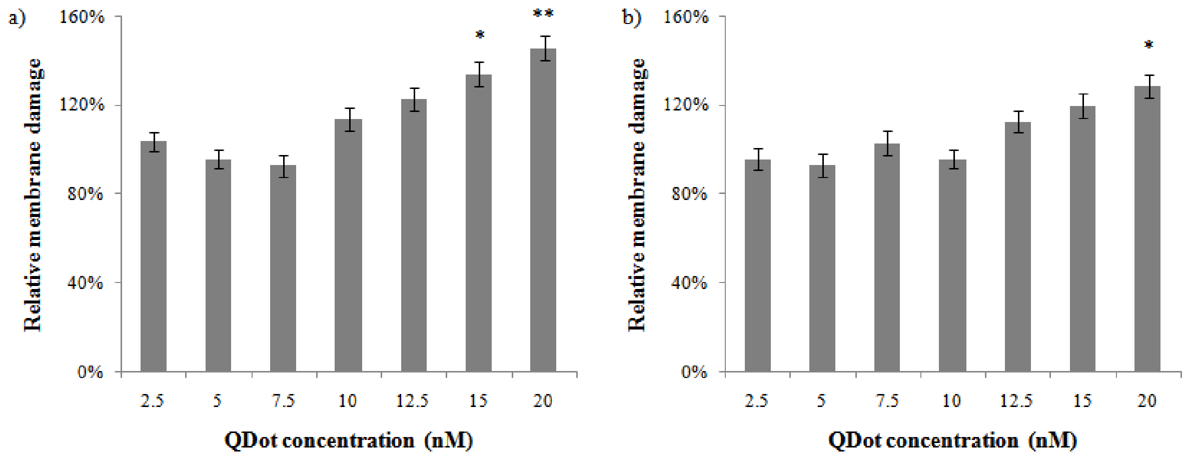

Figure S3. Histograms representing the relative cell membrane damage of (a) MSC and (b) MEF cells exposed to the QDots at 2.5, 5, 7.5, 10, 12.5, 15 or 20 nM for 24 h. Data are shown

as relative values normalized to untreated control cells (= 100%) as mean + SEM (n = 3).The degree of statistical significance is indicated where appropriate (*: $p \leq 0.05$, **: $p \leq 0.01$).

Shown below are the data for cellular LC3 levels.

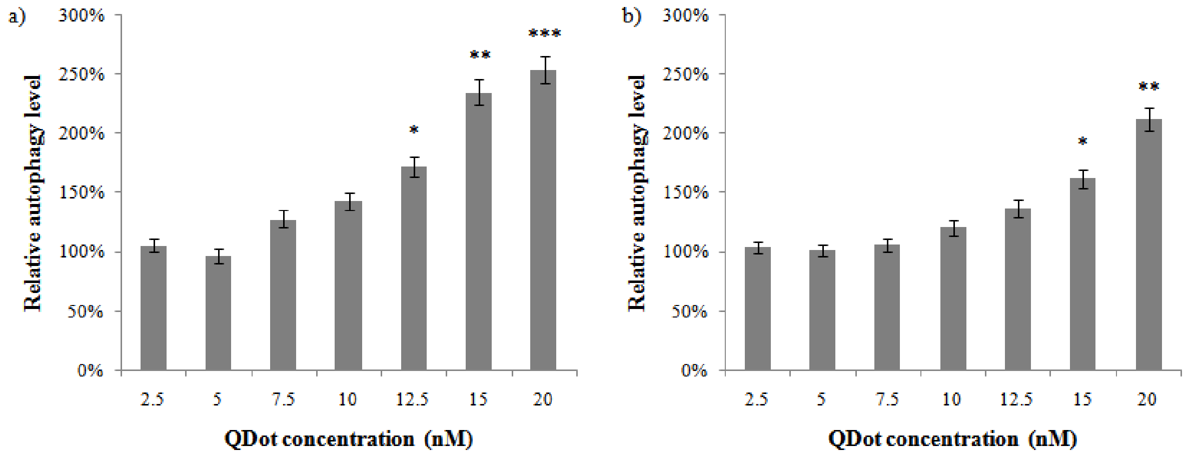

Figure S4. Histograms representing the relative autophagy of (a) MSC and (b) MEF cells exposed to the QDots at 2.5, 5, 7.5, 10, 12.5, 15 or 20 nM for 24 h. Data are shown as relative values normalized to untreated control cells (= 100%) as mean + SEM (n = 3).The degree of statistical significance is indicated where appropriate (*: $p \leq 0.05$, **: $p \leq 0.01$, ***: $p \leq 0.001$).

Shown below are the data for cellular area measurements.

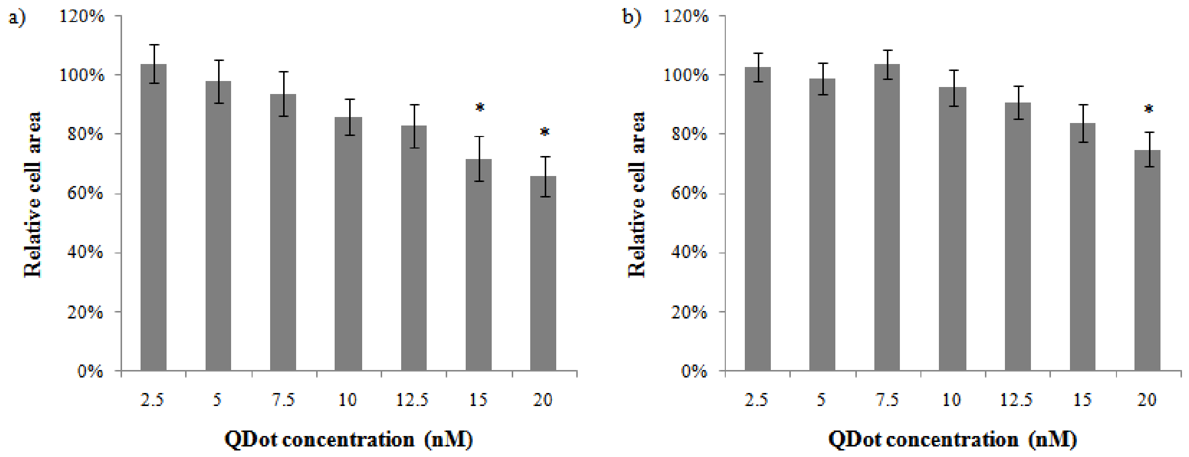

Figure S5. Histograms representing the relative cell area of MSC (left) and MEF (right) cells exposed to the QDots at 2.5, 5, 7.5, 10, 12.5, 15 or 20 nM for 24 h. Data are shown as relative values normalized to untreated control cells (= 100%) as mean + SEM (n = 3).The degree of statistical significance is indicated where appropriate (*: $p \leq 0.05$).

Shown below are the data for cell skewness.

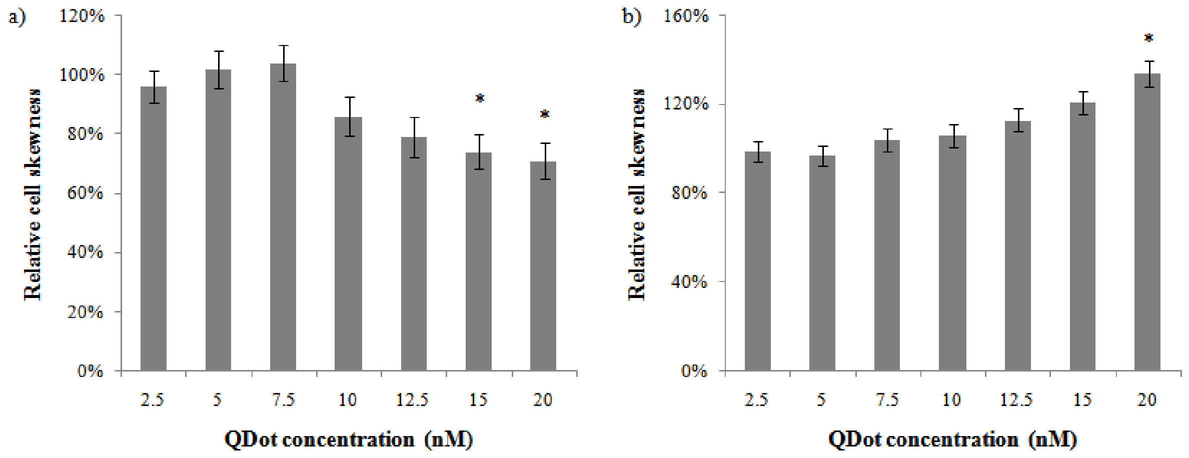

Figure S6. Histograms representing the relative cell skewness of (a) MSC and (b) MEF cells exposed to the QDots at 2.5, 5, 7.5, 10, 12.5, 15 or 20 nM for 24 h. Data are shown as relative values normalized to untreated control cells (= 100%) as mean + SEM (n = 3).The degree of statistical significance is indicated where appropriate (*: $p \leq 0.05$).

Shown below are the data for mitochondrial network size (mito_stress).

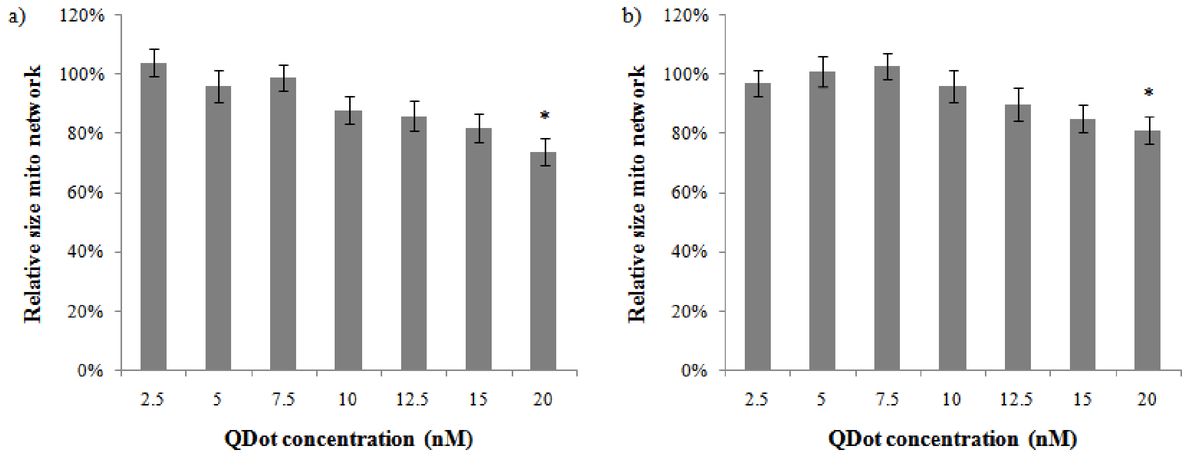

Figure S7. Histograms representing the relative size of the mitochondrial network of (a) MSC and (b) MEF cells exposed to the QDots at 2.5, 5, 7.5, 10, 12.5, 15 or 20 nM for 24 h. Data are shown as relative values normalized to untreated control cells (= 100%) as mean + SEM (n = 3).The degree of statistical significance is indicated where appropriate (*: $p \leq 0.05$).

Shown below are the data for mitochondrial ROS.

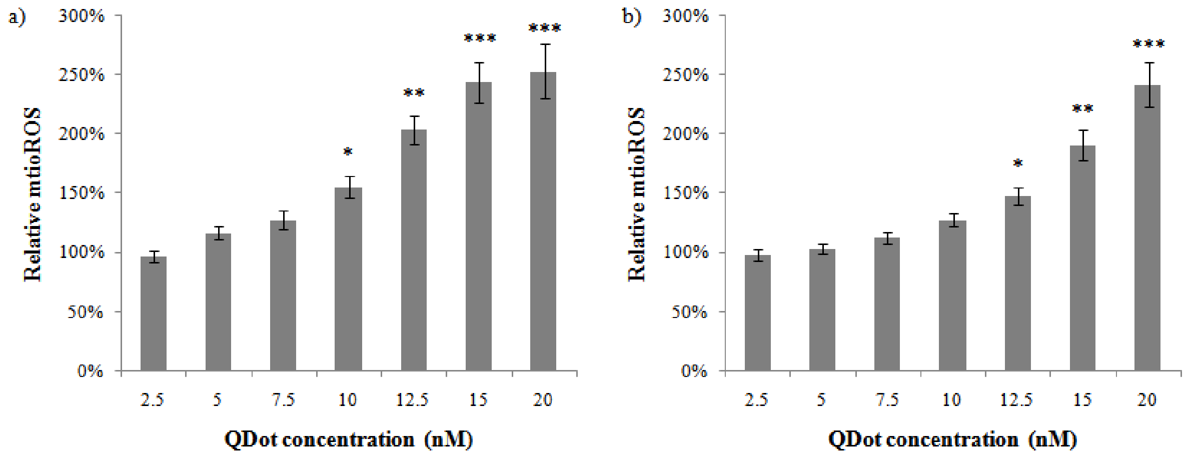

Figure S8. Histograms representing the relative mitochondrial ROS of (a) MSC and (b) MEF cells exposed to the QDots at 2.5, 5, 7.5, 10, 12.5, 15 or 20 nM for 24 h. Data are shown as relative values normalized to untreated control cells (= 100%) as mean $\pm$ SEM (n = 3).The degree of statistical significance is indicated where appropriate (*: $p \leq 0.05$, **: $p \leq 0.01$, ***: $p \leq 0.001$).

Supplementary section 4: Cellular effects linked to cellular quantum dot levels.

Based on the cellular effects of the QDots and the wide distribution of cellular QDots, the experiments were repeated for cells exposed to sub-cytotoxic concentrations of QDots (10 nM for MSCs and 12.5 nM for MEFs). At these concentrations, the cellular effects observed were only minor and non-significant (apart from mitochondrial ROS generation), overall, no clear cytotoxicity was detected. Linking the cellular effects to the cellular QDot concentrations allowed us to study the elicited effects in more detail and enabled us to see whether particular subpopulations (based on cellular QDot levels) displayed a different profile than the total population.

Shown below are the data for mitochondrial size (mito-stress):

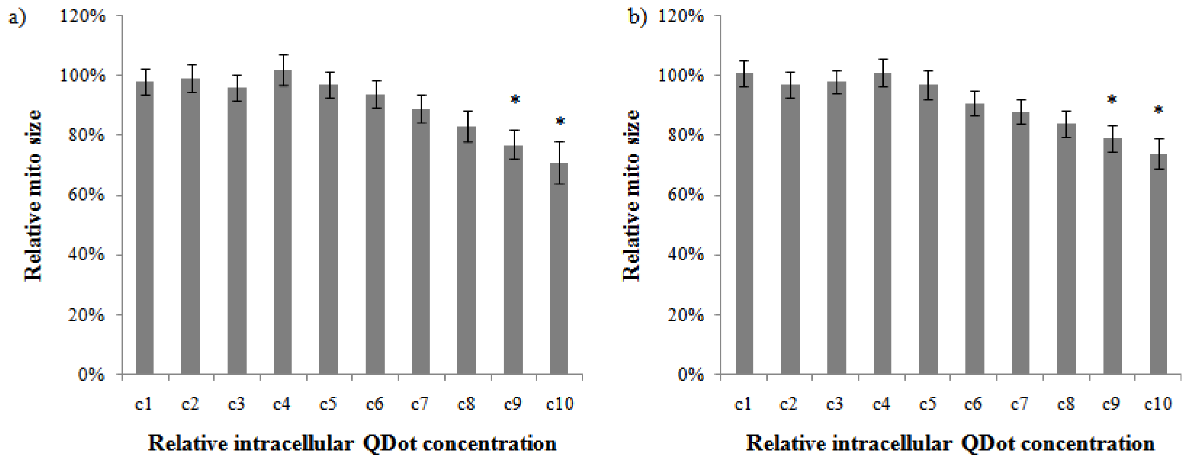

Figure S9. Histograms representing the relative size of the mitochondrial network of (**a**) MSC and (**b**) MEF cells exposed to (**a**) 10 nM and (**b**) 12.5 nM Qdots for 24 h, respectively. Data are expressed in function of the relative cellular QDot levels (c1: lowest, c10: highest) as relative values normalized to untreated control cells (= 100%) as mean + SEM (*n* = 3).The degree of statistical significance is indicated where appropriate (*: p ≤0.05).

Shown below are the data for mitochondrial ROS:

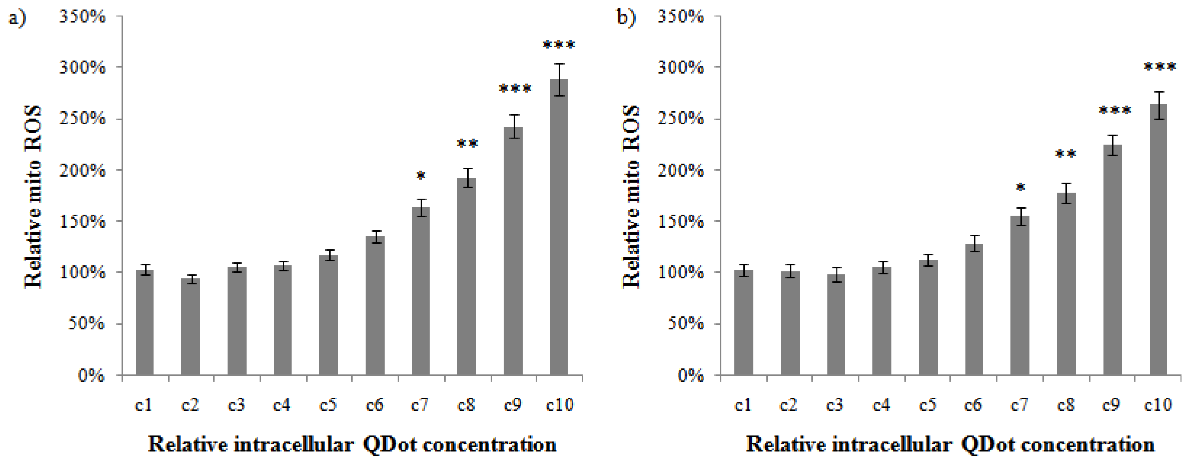

Figure S10. Histograms representing the relative mitochondrial ROS of (**a**) MSC and (**b**) MEF cells exposed to (**a**) 10 nM and (**b**) 12.5 nM Qdots for 24 h, respectively. Data are expressed in function of the relative cellular QDot levels (c1: lowest, c10: highest) as relative values normalized to untreated control cells (= 100%) as mean + SEM (*n* = 3). The degree of statistical significance is indicated where appropriate (*: p ≤0.05, **: p ≤0.01, ***: p ≤ 0.001).

Shown below are the data for cellular area:

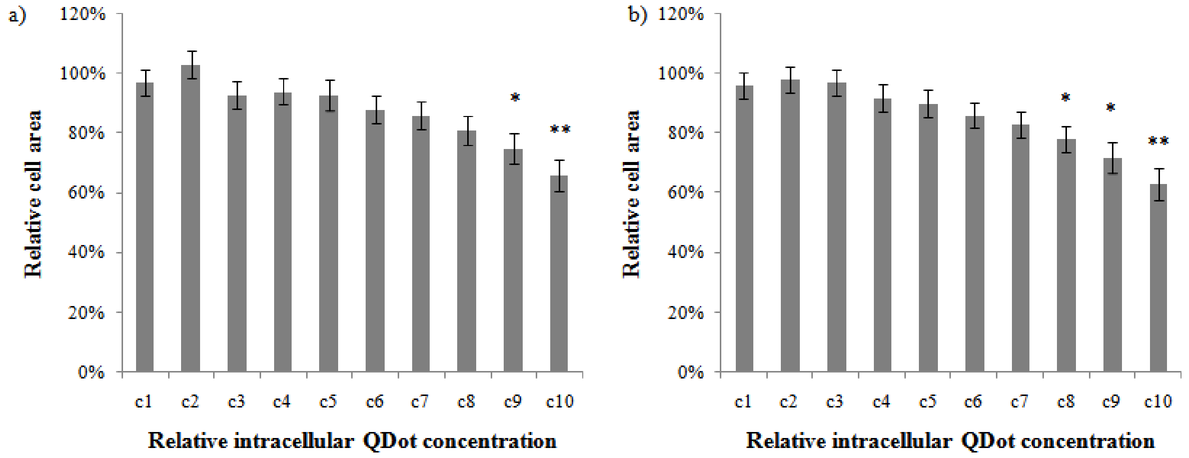

Figure S11. Histograms representing the relative cellular area of (**a**) MSC and (**b**) MEF cells exposed to (**a**) 10 nM and (**b**) 12.5 nM QDots for 24 h, respectively. The data are expressed in function of the relative cellular QDot levels (c1: lowest, c10: highest) as relative values normalized to untreated control cells (= 100%) as mean + SEM (*n* = 3).The degree of statistical significance is indicated where appropriate (*: p ≤ 0.05, **: p ≤ 0.01).

Shown below are the data for cellular skewness:

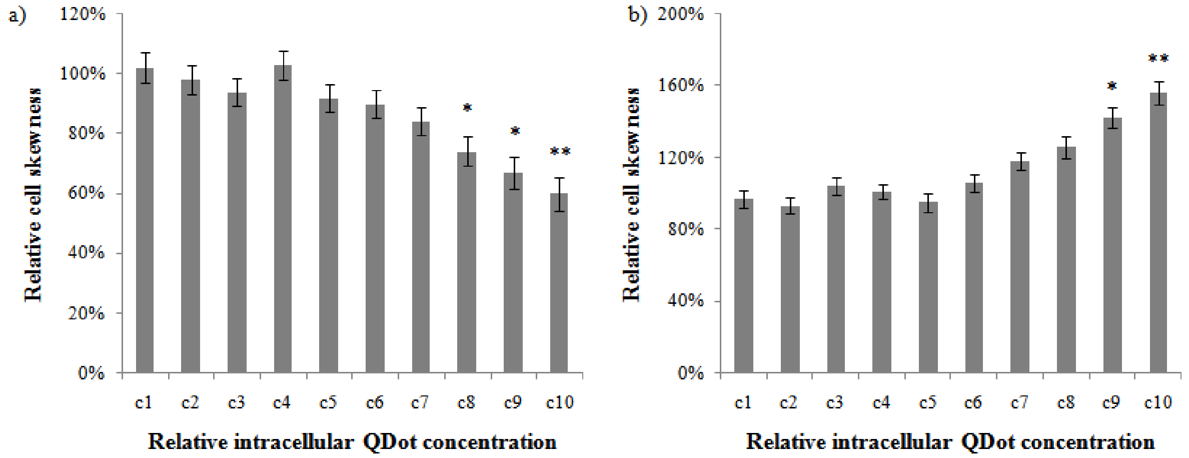

Figure S12. Histograms representing cellular skewness of (**a**) MSC and (**b**) MEF cells exposed to (**a**) 10 nM and (**b**) 12.5 nM QDots for 24 h, respectively. Data are expressed in function of the relative cellular QDot levels (c1: lowest, c10: highest) as relative values

normalized to untreated control cells (= 100%) as mean ± SEM (*n* = 3).The degree of statistical significance is indicated where appropriate (*: p ≤0.05, **: p ≤0.01).

Shown below are the data for cellular LC3 levels:

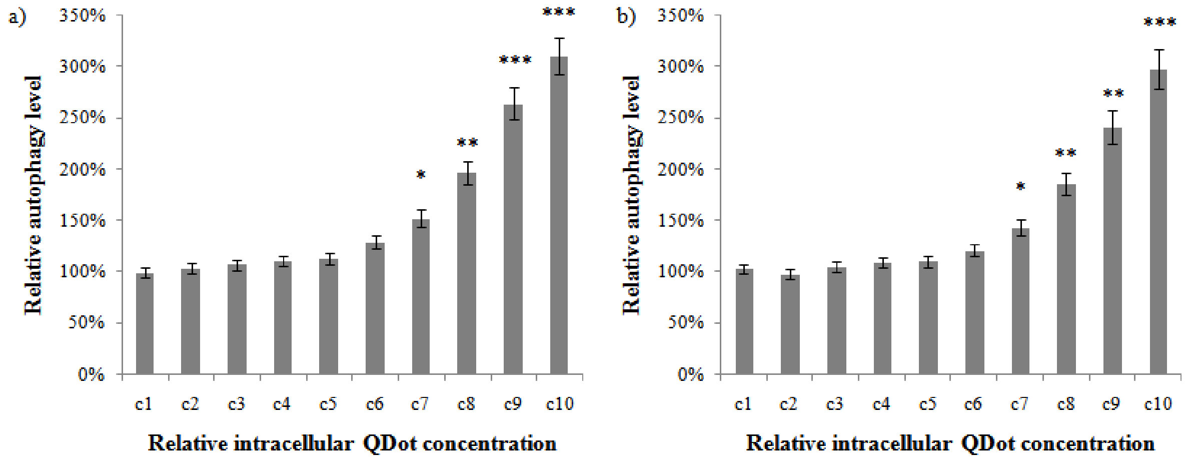

Figure S13. Histograms representing the relative level of cellular autophagy of (**a**) MSC and (**b**) MEF cells exposed to (**a**) 10 nM and (**b**) 12.5 nM Qdots for 24 h, respectively. Data are expressed in function of the relative cellular QDot levels (c1: lowest, c10: highest) as relative values normalized to untreated control cells (= 100%) as mean ± SEM (*n* = 3).The degree of statistical significance is indicated where appropriate (*: p ≤0.05, **: p ≤ 0.01, ***: p ≤ 0.001).

Shown below are the data for cell viability:

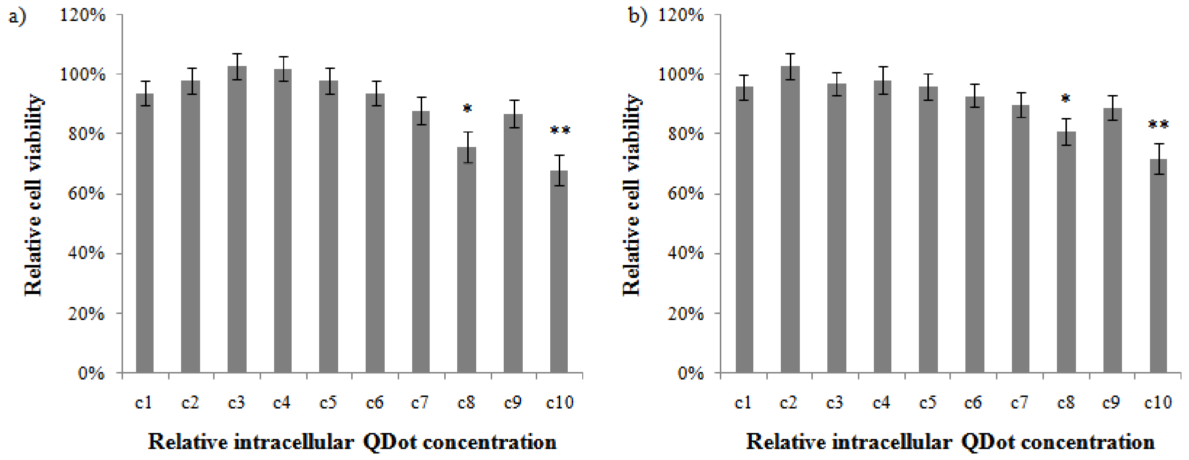

Figure S14. Histograms representing the relative cellular viability of (**a**) MSC and (**b**) MEF cells exposed to (**a**) 10 nM and (**b**) 12.5 nM Qdots for 24 h, respectively. Data are expressed in function of the relative cellular QDot levels (c1: lowest, c10: highest) as relative values normalized to untreated control cells (= 100%) as mean ± SEM (*n* = 3). The degree of statistical significance is indicated where appropriate (*: p ≤0.05, **: p ≤0.01).

Shown below are the data for cellular membrane damage:

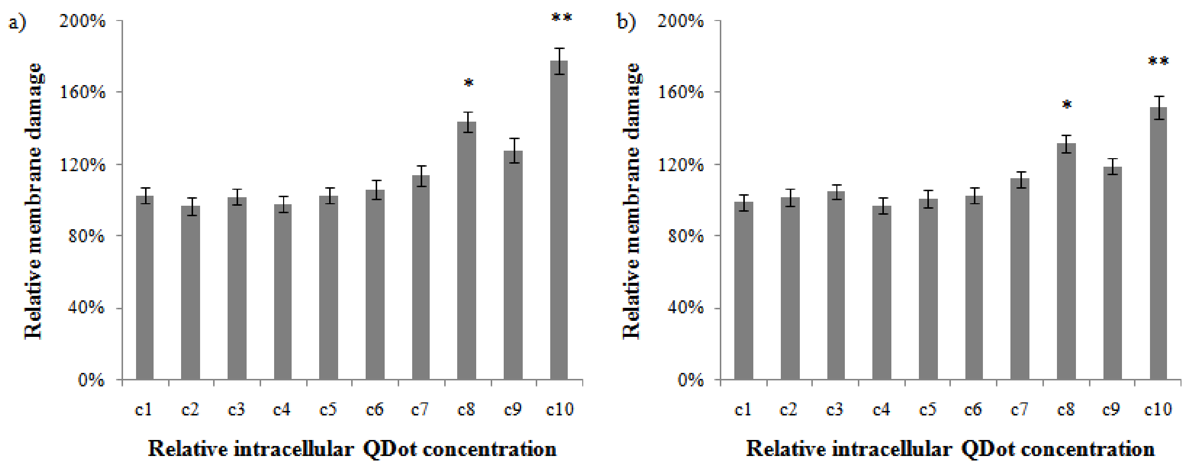

Figure S15. Histograms representing the relative cellular membrane damage of (**a**) MSC and (**b**) MEF cells exposed to (**a**) 10 nM and (**b**) 12.5 nM QDots for 24 h, respectively. Data are expressed in function of the relative cellular QDot levels (c1: lowest, c10: highest) as relative values normalized to untreated control cells (= 100%) as mean $\pm$ SEM (n = 3).The degree of statistical significance is indicated where appropriate (*: $p \leq 0.05$, **: $p \leq 0.01$).

Supplementary section 5: Conflicting cellular response mechanisms

In contrast to all the other parameters studied in this work the effects of the QDots on cell viability and membrane damage showed no clear correlation with cellular QDot levels. This may be due to the cross-talk of different cell survival and cell death pathways which are activated to different levels depending on the cellular QDot levels. As the QDots are shown to induce autophagy and autophagy is known to inhibit apoptosis[1], these mechanisms are further evaluated in detail using the previously described methodology (Supplementary section 5). For this purpose, apoptosis-deficient cells (*i.e.* MEF Bax/Bak DKO cells or MSCs treated with 100 µM Z-VAD-fmk) and autophagy-deficient cells (*i.e.* MEF Atg5 KO or MSCs treated with 1 mM 3-MA) are used.

Shown below are the data for cell viability:

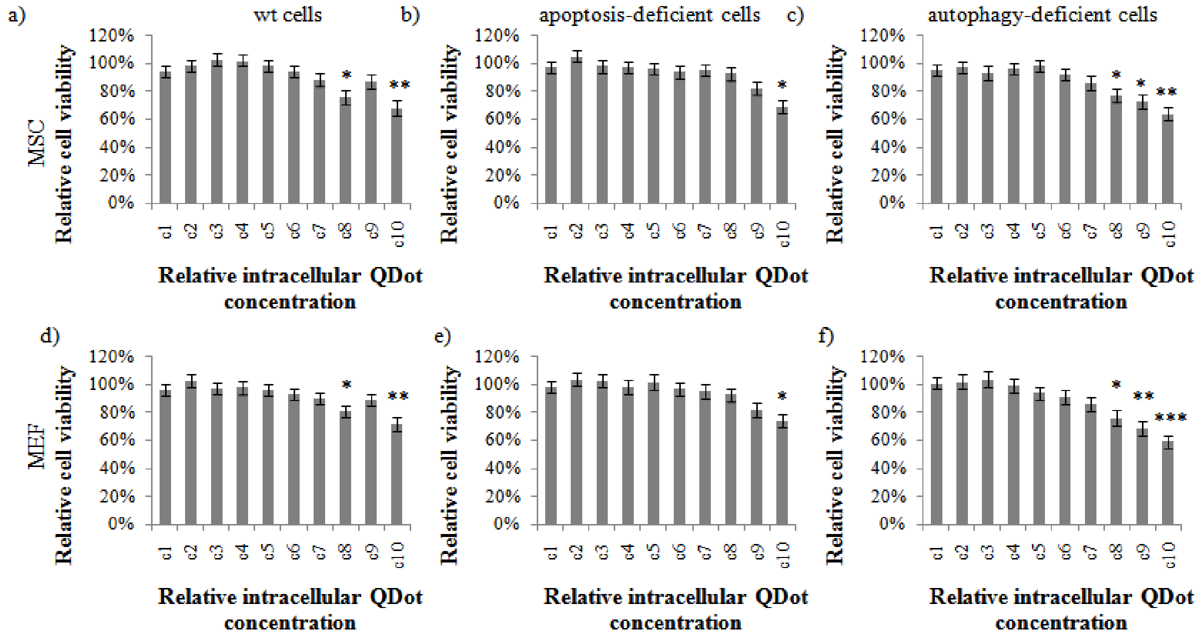

Figure S16. Histograms representing the relative cellular viability of (**a**) MSCs, (**b**) MSCs treated with 100 µM Z-VAD-fmk, (**c**) MSCs treated with 1 mM 3-MA, (**d**) MEFs, (**e**) MEF Bax/Bak DKO cells and (**f**) MEF Atg5 KO cells (**a,b,c**) 10 nM and (**d,e,f**) 12.5 nM Qdots for 24 h, respectively. Data are expressed in function of the relative cellular QDot levels (c1: lowest, c10: highest) as relative values normalized to untreated control cells (= 100%) as mean $\pm$ SEM (n = 3). The degree of statistical significance is indicated where appropriate (*: $p \leq 0.05$, **: $p \leq 0.01$).

Shown below are the data for cellular apoptosis:

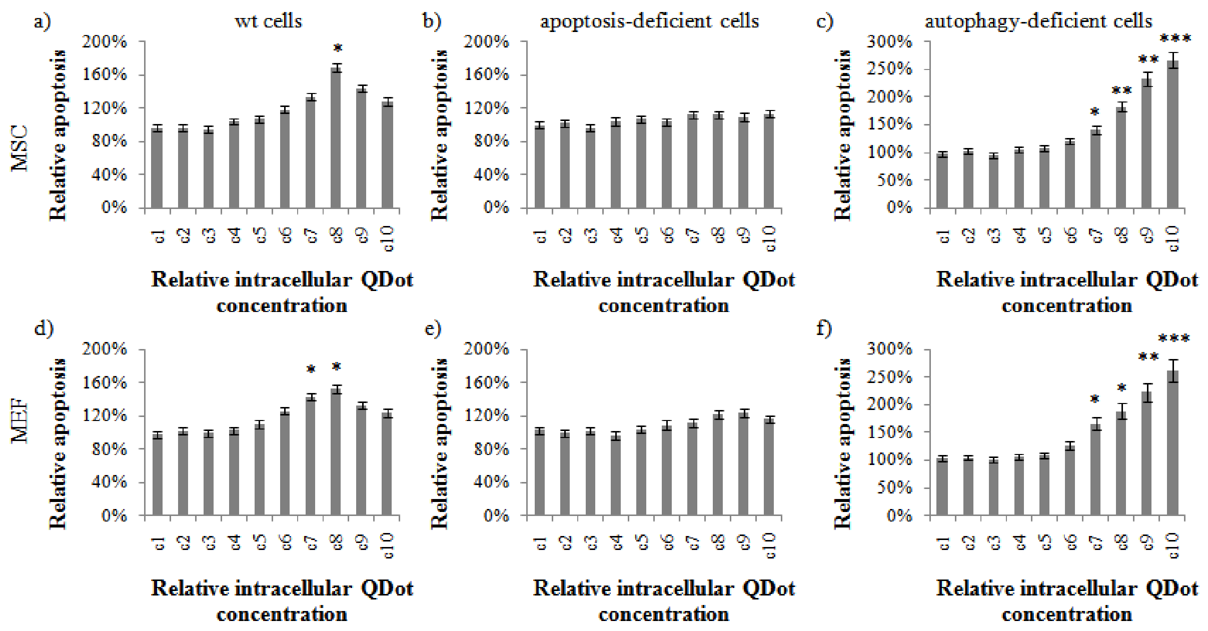

Figure S17. Histograms representing the relative cellular apoptosis of (**a**) MSCs, (**b**) MSCs treated with 100 µM Z-VAD-fmk, (**c**) MSCs treated with 1 mM 3-MA, (**d**) MEFs, (**e**) MEF Bax/Bak DKO cells and (**f**) MEF Atg5 KO cells (**a,b,c**) 10 nM and (**d,e,f**) 12.5 nM Qdots for 24 h, respectively. Data are expressed in function of the relative cellular QDot levels (c1: lowest, c10: highest) as relative values normalized to untreated control cells (= 100%) as mean ± SEM (n = 3). The degree of statistical significance is indicated where appropriate (*: $p \leq 0.05$, **: $p \leq 0.01$).

Shown below are the data for cellular LC3 levels:

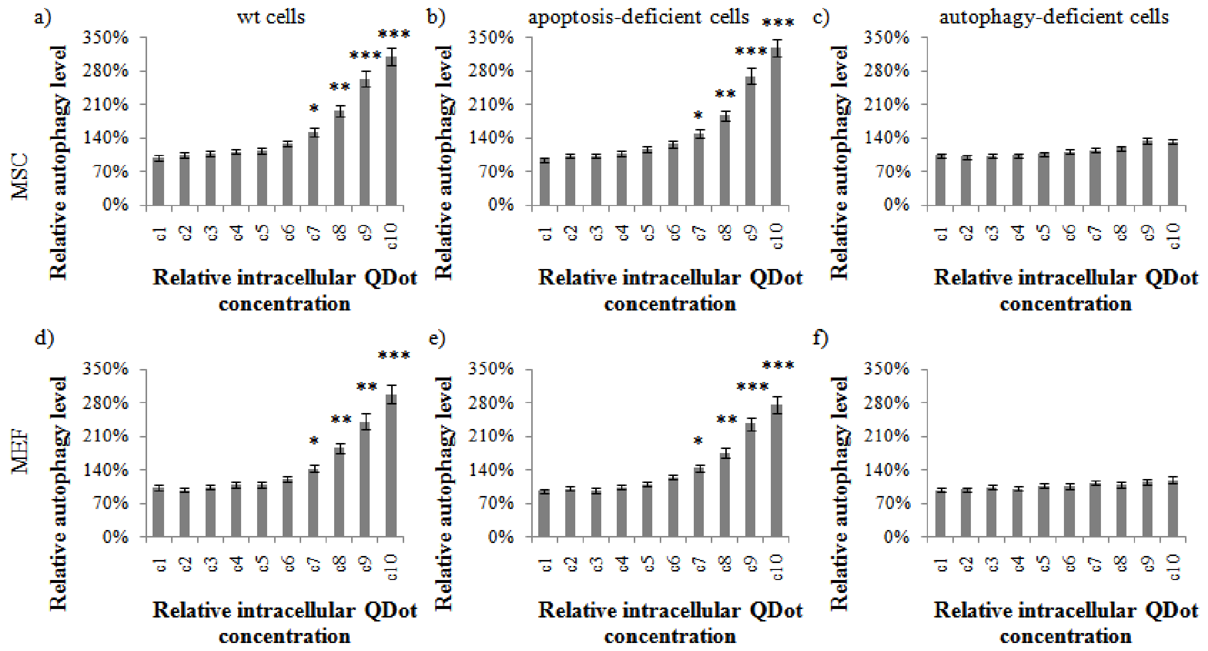

Figure S18. Histograms representing the relative autophagy levels of (**a**) MSCs, (**b**) MSCs treated with 100 µM Z-VAD-fmk, (**c**) MSCs treated with 1 mM 3-MA, (**d**) MEFs, (**e**) MEF

Bax/Bak DKO cells and (**f**) MEF Atg5 KO cells (**a,b,c**) 10 nM and (**d,e,f**) 12.5 nM QDots for 24 h, respectively. Data are expressed in function of the relative cellular QDot levels (c1: lowest, c10: highest) as relative values normalized to untreated control cells (= 100%) as mean $\pm$ SEM (*n* = 3). The degree of statistical significance is indicated where appropriate (*: $p \leq 0.05$, **: $p \leq 0.01$).

The data obtained show a clear and distinct effect of the different inhibitors on their respective mechanisms. Inhibition of apoptosis has no effect on the level of autophagy, whereas inhibition of autophagy renders the cells more prone to apoptosis. Inhibition of apoptosis results in a partial recovery of cell viability at c8 levels, but has no real impact on c10 levels. Inhibition of autophagy results in higher levels of cell death and restores the correlation between cell death and cellular QDot levels. Together, these data show the impact of autophagy, acting as a cytoprotective mechanism in categories 8 and 9, but resulting in direct cell death at the highest levels of cellular QDots (c10).

Materials and methods

High-content imaging of cell-nanoparticle interactions

Cell viability and membrane damage

Following cellular exposure to the QDots, the cells were washed twice with phosphate buffered saline (PBS; Gibco, Invitrogen, Belgium) and treated with 2 µM fixable Live-Dead Green dead cell stain (Molecular Probes, Life Technologies Europe, BV, Belgium) in 250 µl/well of PBS (with Ca^{2+} and Mg^{2+}) and incubated in the dark for 30 min at room temperature. Next, the staining media was aspirated, cells were washedgently with PBS (3x) fixed with 4% paraformaldehyde (PFA) for 15 min at room temperature. The fixative was aspirated and cells were washed three times with PBS. Cells were then counterstained using Hoechst 33342 Nuclear stain (20 µg/ml PBS in 250 µl/well) for 15 min at ambient temperature in the dark. The nuclear counterstain was then removed, cells were washed three times with PBS and 500 µl of PBS was added to every well, after which the plates were analyzed using the InCell 2000 analyzer (GE Healthcare Life Sciences, Belgium). During acquisition, a minimum of 50 fields per well were imaged using a 20x objective for the following channels: UV/blue for Hoechst nuclear stain, FITC/FITC for the Live-Dead Green dead cell stain and FITC/Cy5.5 for the QDots. Data analysis was then performed on the InCell Investigator software (GE Healthcare Life Sciences, Belgium) using in-house developed protocols, using a minimum of 5000 cells/condition. The level of cell viability was calculated as follows: First, cells were segmented based on the Hoechst stain and the perinuclear region was determined by enlarging the nuclear stain 2.5-fold and using the original Hoechst stain images as seed images. For cell viability based on cellular exposure concentrations, cell viability was calculated by determining the number of total cells minus the number of dead cells (dead cells are defined as cells with clear green nuclei, where the intensity is minimally 3-fold above noise level and having an area of minimally 2 µm²). These values were then normalized to control values (100%) to indicate the degree of cell viability.

For membrane damage, the analysis occurred similarly. All green dots in the perinuclear area with a minimum intensity of 3-fold above the noise level and with a size of minimally 0.1 µm² but smaller than 2 µm² were selected. The ratio of this value with the value obtained for control cells was then given to indicate the level of membrane damage.

For cell viability based on intracellular QDot levels, analysis took a few extra steps. Firstly, the level of intracellular QDots was determined using the product of the total cellular area of the QDots multiplied with the total intensity. Based on the values obtained, the cells were subdivided into 10 different categories encompassing the entire cellular range of QDots, ranging from low (category 1) to high (category 10) intracellular QDot levels. Using hierarchical clustering, the level of cell death and membrane damage were analyzed as described above, where the values obtained were linked to the level of cellular QDots, resulting in a histogram of the biological parameter as a function of cellular QDot levels spread over 10 categories.

Oxidative stress

After labeling the cells, they werewashed (3x) with 500 µl PBS/well, after which the cells were incubated with 500 µl full media containing 5 µM CellROX Green (Molecular Probes, Invitrogen, Belgium) for 30 min at 37°C. The staining solution was aspirated, cells were washed with 500 µl PBS/well (3x) and fixed with 4% PFA for 15 min at room temperature. The fixative was removed, cells were washed (3x) with PBS (500 µl/well) after which 500 µl of PBS was added per well and plates were kept at 4°C in a dark container until they were needed for analysis using the InCell 2000 high-content imaging system. For acquisition, the following channels were selected: UV/blue for Hoechst nuclear stain, FITC/FITC for the CellROX Green stain and FITC/Cy5.5 for the QDots.Data analysis was then performed on the InCellInvestigator software (GE Healthcare Life Sciences, Belgium) using in-house developed protocols, using a minimum of 5000 cells/condition. The level of oxidative stress was then calculated as follows: First, cell nuclei were segmented based on the blue channel (Hoechst). As CellROX Green localizes in the nucleus upon oxidation, the intensity of light emitted in the green channel was then determined for every area of the corresponding nuclei. The intensity of every nucleus was then calculated for the green channel and normalized to the intensity level of untreated control cells (100%).

For oxidative stress based on intracellular quantum dot levels, analysis took a few extra steps. Firstly, the total cellular area of QDots was determined by multiplying the area of the nucleus by 2.5-fold for a perinuclear region (where the QDots were localized), using the original Hoechst staining images as seed images for segmentation. Any possible overlapping regions were only counted once. The level of intracellular QDots was determined using the product of the total cellular area of the QDots multiplied with their total intensity. The cell-based QDots were then assigned to the perinuclear cell region. Based on the values obtained, the cells were subdivided into 10 different categories encompassing the entire cellular range of QDots, ranging from low (category 1) to high (category 10) intracellular QDot levels. Using hierarchical clustering, the level of oxidative stress was analyzed as described above, where the values obtained were then linked to the level of cellular QDots, resulting in a histogram of the biological parameter as a function of cellular QDot levels spread over 10 categories.

Mitochondrial health

After labeling the cells, they werewashed (3x) with 500 µl PBS/well, after which the cells were stained in 250 µl PBS (containing Ca^{2+} and Mg^{2+}) containing 200 nM MitoTracker Red CMXRos (Molecular Probes, Invitrogen, Belgium) and incubated for 30 min at room temperature. The staining solution was then aspirated, cells were washed with 500 µl PBS/well (3x) and fixed with 4% PFA for 15 min at room temperature. The fixative was removed, cells were washed (3x) with PBS (500 µl/well) after which 500 µl of PBS was added per well and plates were kept at 4°C in a dark container until they were needed for analysis using the InCell 2000 high-content imaging system. For acquisition, the following channels were selected: UV/blue for Hoechst nuclear stain, DsRed/DsRed for the MitoTracker Red CMXRos stain and FITC/Cy5.5 for the QDots. Data analysis was then performed using the InCell Investigator software (GE Healthcare Life Sciences, Belgium)

using in-house developed protocols, analyzing a minimum of 5000 cells/condition. The mitochondrial stress and ROS werethen calculated as follows: First, cell nuclei were segmented based on the blue channel (Hoechst). Then, the DsRed/DsRed channel was segmented, using the nuclear target channel as seed images. Based on the segmented mitochondrial images, the overall area of cellular mitochondria were calculated, for any dot in the mitochondrial channel that had an intensity of minimum3-fold higher than the background noise level. The total area of cellular mitochondria was determined as a marker for mitochondrial stress, where damaged mitochondria change shape, turning from an elongated to a more spherical morphology[2]. The total area of cellular mitochondria was then normalized to the area of mitochondria in untreated control cells (100%). For mitochondrial ROS, the level of fluorescence intensity of the segmented mitochondria was determined. The intensity of the mitochondrial signal was then normalized to the intensity level of untreated control cells (100%).

For mitochondrial stress and ROS analysis based on intracellular QDot levels, analysis took a few extra steps. Firstly, the total cellular area of QDots was determined by multiplying the area of the nucleus by 2.5-fold for a perinuclear region (where the QDots were localized), using the original Hoechst staining images as seed images for segmentation. Any possible overlapping regions were only counted once. The level of intracellular QDots was determined using the product of the total cellular area of the QDots multiplied with the total intensity. The cell-based QDots were then assigned to the perinuclear cell region. Based on the values obtained, the cells were subdivided into 10 different categories encompassing the entire cellular range of QDots, ranging from low (category 1) to high (category 10) cellular QDot levels. Using hierarchical clustering, the level of mitochondrial stress and ROS were analyzed as described above, where the values obtained were then linked to the level of cellular QDots, resulting in a histogram of the biological parameter as a function of intracellular QDot levels spread over 10 categories.

Cell morphology and skewness

After cellular exposure to the QDots, cells were washed (3x) with 500 µl PBS/well and fixed for 15 min at room temperature with 4% PFA. The fixative was then aspirated, cells were washed (3x) with PBS (500 µl/well) after which cells were permeabilised with 250 µl/well of Triton X-100 (1%) for 10 min at room temperature. Cells were then blocked with 10% serum-containing PBS for 30 min at room temperature. Next, cells were stained using 200 µl of staining solution per well (15 µl of 1/100 dilution of the stock in blocking buffer) of Acti-Stain 555 (Tebu-Bio, Belgium) and incubated for 90min in the dark at room temperature. The staining solution was aspirated, cells were washed (3x) with PBS (500 µl/well) after which 500 µl fresh PBS was added to each well and the plates were kept at 4°C in a dark container until analyzed using the InCell 2000 high-content imaging system. For acquisition, the following channels were selected: UV/blue for Hoechst nuclear stain, DsRed/DsRed for the actin stain and FITC/Cy5.5 for the QDots. Data analysis was then performed on the InCell Investigator software (GE Healthcare Life Sciences, Belgium) using in-house developed protocols, using a minimum of 5000 cells/condition. The size of the cells was calculated as follows: First, cell nuclei were segmented based on the blue channel. Cells were then

segmented using the DsRed channel, where any holes in the cells were filled up and included. Cells on the border of the field of view were excluded from the analysis. The segmentation was based on the blue channel as seed channel for the nucleus. The total area of every individual cell was then determined. For determination of skewness (i.e. the shape of the cells, being the ratio of cell width over cell length), the same approach was used. After segmentation, the “form factor” was calculated which provides the ratio of the cell width over cell length. This value will always be between 0 (straight line) and 1 (perfect circle). For both parameters, the values obtained were then normalized to the values obtained for untreated control cells (100%).

For cell area analysis based on intracellular QDot levels, analysis took a few extra steps. Firstly, the total cellular area of QDots was determined by multiplying the area of the nucleus by 2.5-fold for a perinuclear region (where the QDots were localized), using the original Hoechst staining images as seed images for segmentation. Any possible overlapping regions were only counted once. The level of intracellular QDots was determined using the product of the total cellular area of the QDots multiplied with their total intensity. The cell-based QDots were then assigned to the perinuclear cell region. Based on the values obtained, the cells were subdivided into 10 different categories encompassing the entire cellular range of QDots, ranging from low (category 1) to high (category 10) cellular QDot levels. Using hierarchical clustering, the cell area and cell skewness were then analyzed as described above, where the values obtained were then linked to the level of cellular QDots, resulting in a histogram of the biological parameter as a function of cellular QDot levels spread over 10 categories.

Autophagy

After cellular exposure to the QDots, cells were washed (3x) with 500 µl PBS/well and fixed for 15 min at room temperature with 4% PFA. The fixative was then aspirated, cells were washed (3x) with PBS (500 µl/well) after which cells were permeabilised with 250 µl/well of Triton X-100 (1%) for 10 min at room temperature. Cells were then blocked with 10% serum-containing PBS (blocking buffer) for 30 min at room temperature. Next, cells were stained using 200 µl of staining solution per well consisting out of primary mouse anti-LC3 antibody (1/400 dilution in blocking buffer; Cell Signalling Technologies, Belgium) and incubated for 90min in the dark at room temperature. The primary antibody solution was aspirated, cells were washed (3x) with blocking buffer (500 µl/well) after which 200 µl of secondary AF488-conjugated goat anti-mouse IgG antibody (1/250 dilution in blocking buffer; Molecular Probes, Belgium) was added to each well and plates were incubated in the dark for 60 min at room temperature. Following this, the incubation media was aspirated, cells were washed (3x) with PBS (500 µl/well), after which 500 µl fresh PBS was added to each well and the plates were kept at 4°C in a dark container until analyzed using the InCell 2000 high-content imaging system. For acquisition, the following channels were selected: UV/blue for Hoechst nuclear stain, FITC/FITC for the LC3 stain and FITC/Cy5.5 for the QDots. Data analysis was then performed on the InCell Investigator software (GE Healthcare Life Sciences, Belgium) using in-house developed protocols, using a minimum of 5000 cells/condition.

The level of autophagy was calculated as follows: First, cell nuclei were segmented based on the blue channel. Using the FITC channel, the cell cytoplasm was then selected, and cells were segmented, where any holes in the cells were filled up and included and any cells on the border of the field of view were excluded from the analysis. The segmentation was based on the blue channel as seed channel for the nucleus. Using the original FITC/FITC channel, any green dots having an intensity of minimum twice that of the noise level and that were localized within the cytoplasm were segmented, where multiple green dots could be localized within a single cell cytoplasm. Then, the cellular intensity of the green channel was measured for every cell, after which this value was normalized to the control value (100%).

For cell area analysis based on intracellular QDot levels, analysis took a few extra steps. Firstly, the total cellular area of QDots was determined by multiplying the area of the nucleus by 2.5-fold for a perinuclear region (where the QDots were localized), using the original Hoechst staining images as seed images for segmentation. Any possible overlapping regions were only counted once. The level of intracellular QDots was determined using the product of the total cellular area of the QDots multiplied with their total intensity. The cell-based QDots were then assigned to the perinuclear cell region. Based on the values obtained, the cells were subdivided into 10 different categories encompassing the entire cellular range of QDots, ranging from low (category 1) to high (category 10) intracellular QDot levels. Using hierarchical clustering, the cellular autophagy levels were then analyzed as described above, where the values obtained were then linked to the level of cellular QDots, resulting in a histogram of the level of autophagy as a function of cellular QDot levels spread over 10 categories.

Apoptosis (caspase activation)

After cellular exposure to the QDots, cells were washed (3x) with 500 µl PBS/well and fixed for 15 min at room temperature with 4% PFA. The fixative was then aspirated, cells were washed (3x) with PBS (500 µl/well) after which cells were permeabilised with 250 µl/well of Triton X-100 (1%) for 10 min at room temperature. Cells were then blocked with 10% serum-containing PBS (blocking buffer) for 30 min at room temperature. Next, cells were stained using 200 µl of staining solution per well consisting of primary mouse anti-active caspase 3 antibody (1/400 dilution in blocking buffer; Cell Signalling Technologies, Belgium) and incubated for 90min in the dark at room temperature. The primary antibody solution was aspirated, cells were washed (3x) with blocking buffer (500 µl/well) after which 200 µl of secondary AF488-conjugated goat anti-mouse IgG antibody (1/250 dilution in blocking buffer; Molecular Probes, Belgium) was added to each well and plates were incubated in the dark for 60 min at room temperature. Following this, the incubation media was aspirated, cells were washed (3x) with PBS (500 µl/well), after which 500 µl fresh PBS was added to each well and the plates were kept at 4°C in a dark container until analyzed using the InCell 2000 high-content imaging system. For acquisition, the following channels were selected: UV/blue for Hoechst nuclear stain, FITC/FITC for the active caspase 3 stain and FITC/Cy5.5 for the QDots. Data analysis was then performed on the InCell Investigator software (GE Healthcare Life Sciences, Belgium) using in-house developed protocols, using a minimum of 5000 cells/condition.

The level of apoptosis was calculated as follows: First, cell nuclei were segmented based on the blue channel.The cellular perinuclear region was determined by multiplying the area of the nucleus by 2.5-fold. The segmentation was based on the blue channel as seed channel for the nucleus. Any green dots having an intensity of minimum twice that of the noise level and that were localized within the cytoplasm were segmented, where multiple green dots could be localized within a single cell cytoplasm.Then, the cellular intensity of the green channel was measured for every cell, after which this value was normalized to the control value (100%).

For cell area analysis based on intracellular QDot levels, analysis took a few extra steps. The level of intracellular QDots was determined using the product of the total cellular area of the QDots multiplied with their total intensity. The cell-based QDots were then assigned to the perinuclear cell region. Based on the values obtained, the cells were subdivided into 10 different categories encompassing the entire cellular range of QDots, ranging from low (category 1) to high (category 10) intracellular QDot levels. Using hierarchical clustering, the cellular apoptosis levels were then analyzed as described above, where the values obtained were then linked to the level of cellular QDots, resulting in a histogram of the level of apoptosis as a function of cellular QDot levels spread over 10 categories.

Heat map generation

The generation of heat maps was performed using conditional formatting of Excel sheets after z-normalization of all the data. Data are expressed as the fold increase of a certain parameter (cell death, membrane damage, mitochondrial ROS, mitochondrial stress, cell area, cell skewness or autophagy induction) compared to the control levels where the level of increase is indicated by colour-codes.

Confocal microscopy

Endosomal localization of QDots

To assess cellular internalization of the carboxylated QDots, MSC or MEF cells were grown in 25 cm² culture flasks at $2x10^5$ cells/flask. Cells were transduced overnight with CellLight lysosomes-GFP (Molecular Probes, Belgium) according to the manufacturer's instructions. The induction media was then aspirated, cells were lifted with trypsin and seeded in Nunc 4-well glass bottom trays (Nunc) at 7500 cells/well after which the cells were allowed to attach overnight in a humidified atmosphere at 37°C and 5% CO_2. Cells were then incubated with 500 µl growth medium containing 10 nMQDots (for MSCs) or 12.5 nMQDots (for MEFs) for 24 h, after which the incubation media were aspirated, cells were washed (3x) with PBS and fixed with 4% PFA for 15 min at room temperature. Cells were then washed (3x) with PBS after which 500 µl fresh PBS was added to each well and the plates were kept at 4°C in a dark container until analysis using a Nikon A1R Eclipse confocal microscope.

Cellular internalization of QDots

To assess cellular internalization of the carboxylatedQDots, MSC or MEF cells were seeded in Nunc 4-well glass bottom trays (Nunc) at 7500 cells/well after which the cells were allowed to attach overnight in a humidified atmosphere at 37°C and 5% CO_2. The cells were

then incubated with 500 µl growth medium containing 10 nM QDots (for MSCs) or 12.5 nM QDots (for MEFs) for 24 h. The incubation media was then aspirated, cells were washed (3x) with PBS (500 µl/well) and fixed with 4% PFA for 15 min at room temperature, followed by 10 min permeabilization with 1% Triton X-100. The permeabilization media was removed, cells washed (3x) with PBS and blocking buffer (10% serum in PBS) was added after which the cells were incubated for 30 min at room temperature. The blocking media was removed and 200 µl of fresh blocking buffer containing primary mouse anti-α-tubulin antibody (1 µg/ml; Invitrogen) was added per well after which the cells were incubated for 90 min at room temperature in the dark. The primary antibody solution was aspirated, cells were washed (3x) with blocking buffer (500 µl/well) after which 200 µl of secondary AF488-conjugated goat anti-mouse IgG antibody (1/250 dilution in blocking buffer; Molecular Probes, Belgium) was added to each well and plates were incubated in the dark for 60 min at room temperature. Following this, the incubation media was aspirated, cells were washed (3x) with PBS (500 µl/well), after which 500 µl fresh PBS was added to each well and the plates were kept at 4°C until analysis using a Nikon A1R Eclipse confocal microscope.

Statistical analysis

All data are expressed as the mean ± standard error to the mean (SEM). For all experiments, any statistical significance between a single condition and untreated control cells were analyzed using the *t*-test statistical method.

Supplementary references.

1. Maiuri, M.C., Zalckvar, E., Kimchi, A., Kroemer, G. Self-eating and self-killing: crosstalk between autophagy and apoptosis. *Nat. Rev. Mol. Cell. Bio*. **8**, 741-752 (2007).

2. Suen, D.F., Norris, K.F., Youle, R.J. Mitochondrial dynamics and apoptosis. *Genes & Dev*. **22**, 1577-1590. (2008)
